# Physiological and clinical effects of trunk inclination adjustment in patients with respiratory failure: a scoping review and narrative synthesis

**DOI:** 10.1186/s13054-024-05010-1

**Published:** 2024-07-09

**Authors:** Martín H. Benites, Marcelo Zapata-Canivilo, Fabian Poblete, Francisco Labbe, Romina Battiato, Andrés Ferre, Jorge Dreyse, Guillermo Bugedo, Alejandro Bruhn, Eduardo L. V. Costa, Jaime Retamal

**Affiliations:** 1 Unidad de Pacientes Críticos, Clínica Las Condes, Santiago, Chile; 2 Facultad de Medicina, Escuela de Medicina, Universidad Finis Terrae, Santiago, Chile; 3 Doctorado en Ciencias Médicas, Escuela de Medicina, Pontificia Universidad Católica de Chile, Santiago, Chile; 4 Division of Critical Care, Department of Medicine, McMaster University, Hamilton, Canada; 5 Magíster em Bioestadística, Escuela de Salud Pública, Universidad de Chile, Santiago, Chile; 6 Departamento de Medicina Intensiva, Hospital Clínico Pontificia Universidad Católica de Chile, Santiago, Chile; 7 Hospital das Clínicas da Faculdade de Medicina da Universidade de São Paulo, Laboratório de Pneumologia LIM-09, Disciplina de Pneumologia, Heart Institute (Incor), São Paulo, Brazil; 8 Hospital Sírio-Libanês, Research and Education Institute, São Paulo, Brazil

**Keywords:** Acute respiratory distress syndrome, Body position, End-expiratory lung volume, Driving pressure, Respiratory dead space, Trunk inclination, Tidal volume

## Abstract

**Background:**

Adjusting trunk inclination from a semi-recumbent position to a supine-flat position or vice versa in patients with respiratory failure significantly affects numerous aspects of respiratory physiology including respiratory mechanics, oxygenation, end-expiratory lung volume, and ventilatory efficiency. Despite these observed effects, the current clinical evidence regarding this positioning manoeuvre is limited. This study undertakes a scoping review of patients with respiratory failure undergoing mechanical ventilation to assess the effect of trunk inclination on physiological lung parameters.

**Methods:**

The PubMed, Cochrane, and Scopus databases were systematically searched from 2003 to 2023. Interventions: Changes in trunk inclination. Measurements: Four domains were evaluated in this study: 1) respiratory mechanics, 2) ventilation distribution, 3) oxygenation, and 4) ventilatory efficiency.

**Results:**

After searching the three databases and removing duplicates, 220 studies were screened. Of these, 37 were assessed in detail, and 13 were included in the final analysis, comprising 274 patients. All selected studies were experimental, and assessed respiratory mechanics, ventilation distribution, oxygenation, and ventilatory efficiency, primarily within 60 min post postural change.

**Conclusion:**

In patients with acute respiratory failure, transitioning from a supine to a semi-recumbent position leads to decreased respiratory system compliance and increased airway driving pressure. Additionally, C-ARDS patients experienced an improvement in ventilatory efficiency, which resulted in lower PaCO_2_ levels. Improvements in oxygenation were observed in a few patients and only in those who exhibited an increase in EELV upon moving to a semi-recumbent position. Therefore, the trunk inclination angle must be accurately reported in patients with respiratory failure under mechanical ventilation.

**Supplementary Information:**

The online version contains supplementary material available at 10.1186/s13054-024-05010-1.

## Background

Adjusting the trunk inclination from a semi-recumbent head-up position to a supine-flat position or vice versa can produce significant physiological effects on the respiratory system relevant to daily clinical practice [1–3]. These changes in trunk angle have been shown to influence respiratory mechanics [1], chest wall elastance [4], oxygenation [2], end-expiratory lung volume (EELV) [2], and partial pressure of carbon dioxide (PaCO_2_) [1–3]. However, the complete extent of these effects is not yet fully understood and remains a subject of investigation.

Therefore, we conducted a comprehensive scoping review to consolidate the current fragmented knowledge on the influence of trunk inclination on pulmonary physiology. This approach systematically maps the existing research on the subject to provide a more complete picture of the current state of knowledge. Our primary research question focused on exploring how the current literature elucidates the influence of trunk inclination adjustment on lung physiology in mechanically ventilated patients with respiratory failure. This review aimed to summarize the latest knowledge, specifically emphasizing the influence of trunk inclination on respiratory mechanics, ventilation distribution, oxygenation, and ventilatory efficiency in this patient population.

## Methods

This scoping review adhered to the Preferred Reporting Items for Systematic Reviews and Meta-Analyses extension for Scoping Reviews (PRISMA-ScR) guidelines. We have included a checklist of the PRISMA-ScR guidelines in Additional file 1. The Discussion section provides a detailed analysis of the results obtained in this study by an expert panel on acute respiratory failure. This scoping review did not require registration with Prospero. Ethical approval was not required for this study. The authors conceptualized the review objectives and search strategies.

The study criteria focused on adults aged 18 years and older, who experienced respiratory failure, who were invasively ventilated in the ICU, and who had less than seven days of mechanical ventilation. Eligibility required studies to involve adjustments to trunk inclination in the supine position, specifically in the semi-recumbent, reverse Trendelenburg, or supine-flat positions. Suitable studies have employed experimental models or a repeated-measures design, allowing patients to serve as their own controls, and investigated measures of respiratory mechanics, ventilatory efficiency, oxygenation parameters, end-expiratory lung volume, and ventilation distribution, with assessments potentially using electrical impedance tomography. The inclusion criteria were limited to studies on human participants published in any language between 2003 and 2024. Conversely, the exclusion criteria were studies without statistical lung function comparisons across different positions, patients undergoing surgery in operating rooms, patients on venovenous ECMO, patients breathing spontaneously during mechanical ventilation, and studies exclusively focusing on the Trendelenburg position and prone position. Additionally, conference abstracts, unpublished materials, case reports, gray literature, observational studies, and systematic or narrative reviews were not considered.

### Information sources

The following bibliographic databases were searched between 2003 and March 2024 to identify potentially relevant documents: PubMed, Cochrane, and Scopus. The search strategies were drafted by a librarian at Clínica Las Condes and further refined through team discussions. Duplicates were removed by MB and RB.

### Search strategy

The search strategy employed a blend of free-text terms, integrating select Medical Subject Headings (MeSH) based on the PICO´s research question. This approach allowed for a comprehensive and tailored search, ensuring that all relevant areas of inquiry were thoroughly explored to obtain a well-rounded understanding of the subject matter [5]. The detailed retrieval strategy is provided in the Additional file 1.

A limited search strategy was chosen to identify studies that reported physiological results through experimental research. This choice was based on the intention to unveil the physiological effects generated by alterations in chest inclination, thereby ensuring that the research specifically focused on the most relevant and crucial aspects of this variable.

### Data selection and charting process

Initially, we screened titles and abstracts, to remove duplicate entries. Each investigator then performed a more detailed evaluation to shortlist relevant records. In a subsequent in-person meeting, the investigators reviewed the remaining articles, which were included in the scoping review.

The extracted data included publication details, such as the author and publication date, and study-specific details, such as the research design and assessments. Given the broad interpretation of 'trunk inclination' in patients with respiratory failure, our search was expanded using backwards citation tracking of reference lists of the selected articles.

### Data extraction

Two examiners independently extracted the data using the form designed for this scoping review. Subsequently, a third inspector verified the extracted data to ensure its accuracy and completeness. The screening and data extraction procedures were collectively reviewed through discussion. We conducted sequential assessments of the titles, abstracts, and full texts of all publications identified using our search criteria to gauge their potential relevance. Any disagreements concerning study selection or data extraction were resolved through additional discussion.

The data extracted from the included studies included information such as the first author, publication year, study design, participant characteristics and quantity, interventions, outcomes, and conclusions.

### End points

Explore the physiological effects, which include respiratory mechanics, oxygenation, ventilation, and ventilation distribution, induced by changes in trunk inclination.

### Statistical analysis

The mean and standard deviation (SD) of the respiratory system compliance, PaO_2_/FiO_2_, and PaCO_2_ were recorded. The treatment effect is represented by the difference in the means between the two positions. The standard error (SE) indicates the uncertainty of estimating treatment effects. The weight (common/random) of each study and the calculation of the overall effect were recorded using the fixed-effects model (common) and random effects (random). Confidence intervals (CI): IV, fixed + random; 95% CI, 95% confidence interval for the mean difference in respiratory system compliance, PaO_2_/FiO_2_, and PaCO_2_ in both models. Tau^2^, Chi^2^, and I^2^ indicate the degree of heterogeneity between studies, where I^2^ = % indicates the percentage of heterogeneity of the analyzed studies. General effect tests were carried out: common effect: Z = (*p* = value), to analyze whether the difference in means is statistically significant according to the fixed effects model. Random effects: t = (*p* = value) to analyze a statistically significant effect according to the random-effects model.

### Quality assessment

The risk of bias for the included studies was evaluated using the NHLBI Quality Assessment Tool for Before-After (pre-post) Studies with No Control Group. This comprehensive tool, composed of 12 key questions, was chosen for its thoroughness in evaluating aspects such as the clarity of objectives, appropriateness of statistical analyses, and management of confounding variables. Two authors independently assessed each study using this framework to ensure objectivity and depth during the evaluation process. Any scoring discrepancies were resolved through discussion or consultation with a third researcher to ensure a consensus. Based on this meticulous approach, studies were systematically categorized into low, moderate, or high risk of bias tiers, aligning with standardized criteria that reflect their findings' methodological soundness and reliability (Additional file 2).

## Results

### Study selection and study characteristics

**Fig. 1:**
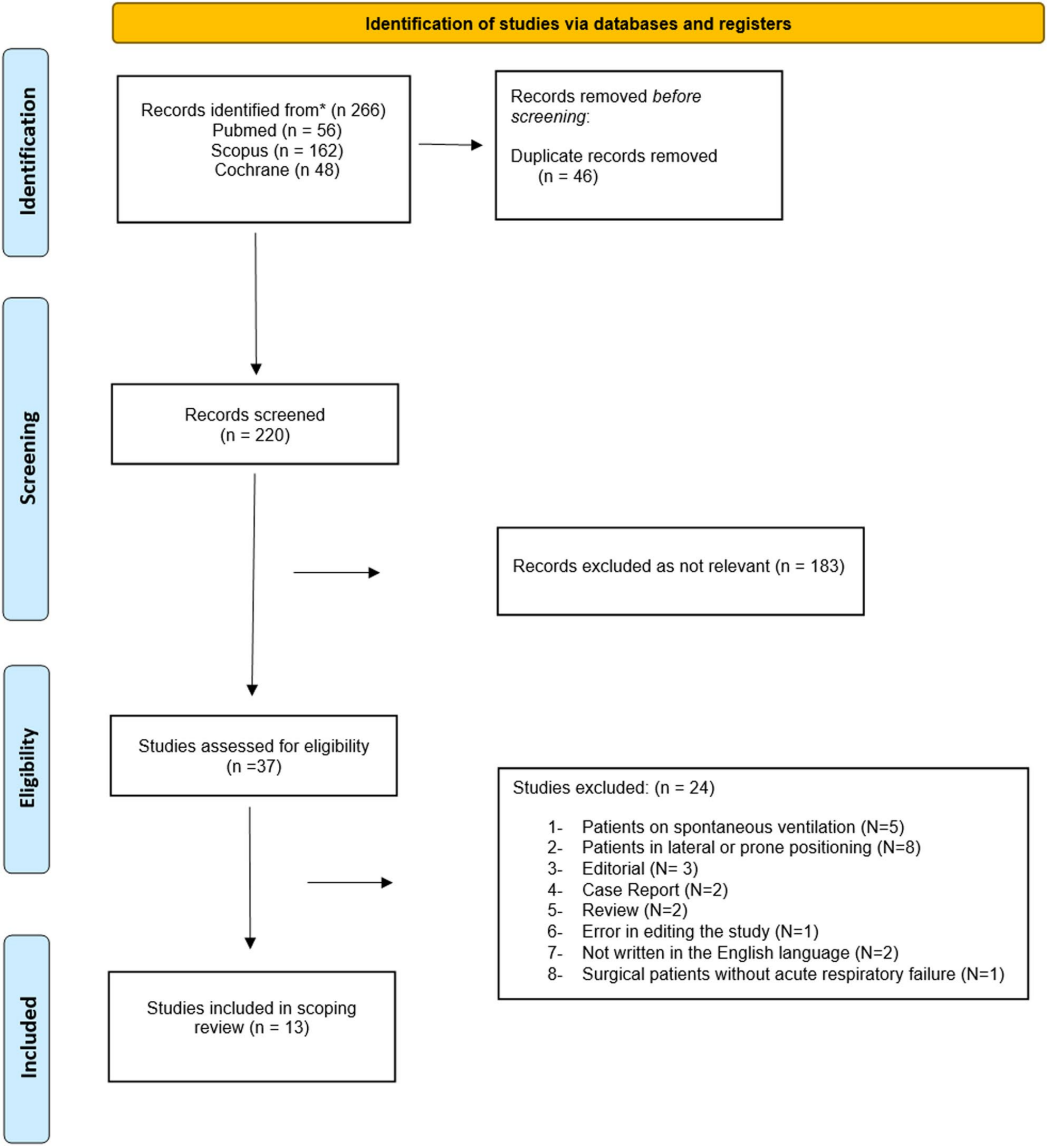
Flow chart

After completing the search for studies and removing all duplicates from the three databases (PubMed, Cochrane, and Scopus), 265 studies were identified. Of these, 37 studies underwent a detailed eligibility assessment and 13 were ultimately included for analysis in this scoping review. The final review included 274 patients [1–3, 6–15]. The flow chart of the study selection process is shown in Figure 1.

All 13 studies included were experimental, repeated measures, and before-after studies conducted at single centers. Of the 13 studies, 12 assessed outcomes related to respiratory mechanics [1–3, 6–11, 13–15], eight evaluated the EELV and ventilation distribution [1–3, 6, 11, 12, 15], seven analysed the effects on oxygenation [1–3, 6, 10, 11, 14], and six scrutinized the impacts on PaCO_2_ and ventilatory efficiency/inefficiency [1–3, 10, 11, 14]. Of these, only one study used volumetric capnography to assess the effect on dead space [3]. In 12 studies, outcomes were assessed within 60 minutes following postural change [1–3, 6, 8–14]. Only one study extended the assessment outcomes 12 hours after postural adjustment [7]. In this evaluation timeframe, neither significant hemodynamic changes nor gastric regurgitation was observed after adjusting for trunk inclination in any of the studies [1–3, 6–15]. Comprehensive details and data for these studies are provided in Additional file 1.

### Characteristics of the sources of evidence

Thirteen experimental studies (100%) were identified. Five studies employed randomized sequences to determine the semi-recumbent and supine-flat position assessment order [1, 10, 11, 14, 15]. The median number of enrolled patients across all studies was 19 (interquartile range [IQR] 16–22). All studies evaluated patients with acute respiratory failure (ARF), within diverse contexts: seven studies focused on patients with classic acute respiratory distress syndrome (ARDS) [2, 3, 6, 7, 9, 14, 15], five dealt with COVID-19 patients [1, 3, 8, 10, 11], one was conducted after cardiac surgery [12], and two studies involved critically ill, morbidly obese patients undergoing mechanical ventilation [13, 14]. In all cases, the patients presented with respiratory failure characterized by a PaO_2_ over the fraction of inspired oxygen (PaO_2_/FIO_2_) < 300 mmHg and were passively ventilated under deep sedation.

### Risk of bias assessment

Of the 13 studies examined, 10 were determined to have a moderate risk of bias [2, 6–12, 14], and the other 3 exhibited a low risk [1, 3, 12]. Nine studies failed to provide calculations of their sample sizes [6–11, 13, 14], and 12 indicated a risk of bias in specifying and defining their study outcomes (Additional file 2) [1, 2, 6–14].

### Expert panel analyses

The authors synthesized the findings from the selected studies, which were then reviewed by an expert committee on respiratory failure and mechanical ventilation (Additional file 1). The primary outcomes of each study were organized into four key categories.

- **Effects on respiratory mechanics and ventilation distribution**
  - Respiratory Mechanics
  - EELV and Ventilation Distribution
- **Effects on blood gas exchange**
  - Oxygenation
  - PaCO_2_ and Ventilatory Efficiency.

1. Effect of trunk inclination adjustment on respiratory mechanics

Ten studies assessed the effect of bed inclination on respiratory mechanics in 254 patients. Of these, ten focused on patients with ARDS (n= 156) [2, 3, 6, 7, 9, 14, 15] and COVID-19-ARDS (C-ARDS, n=82) [1, 3, 8, 11], while two targeted critically ill morbidly obese patients with ARF (n= 36) [13, 14]. The main result was that changing from a supine to a semi-recumbent position resulted in a decrease in respiratory system compliance (C_RS_) and an increase in airway driving pressure. The primary findings are summarized in the Additional file 1.

**Fig. 2:**
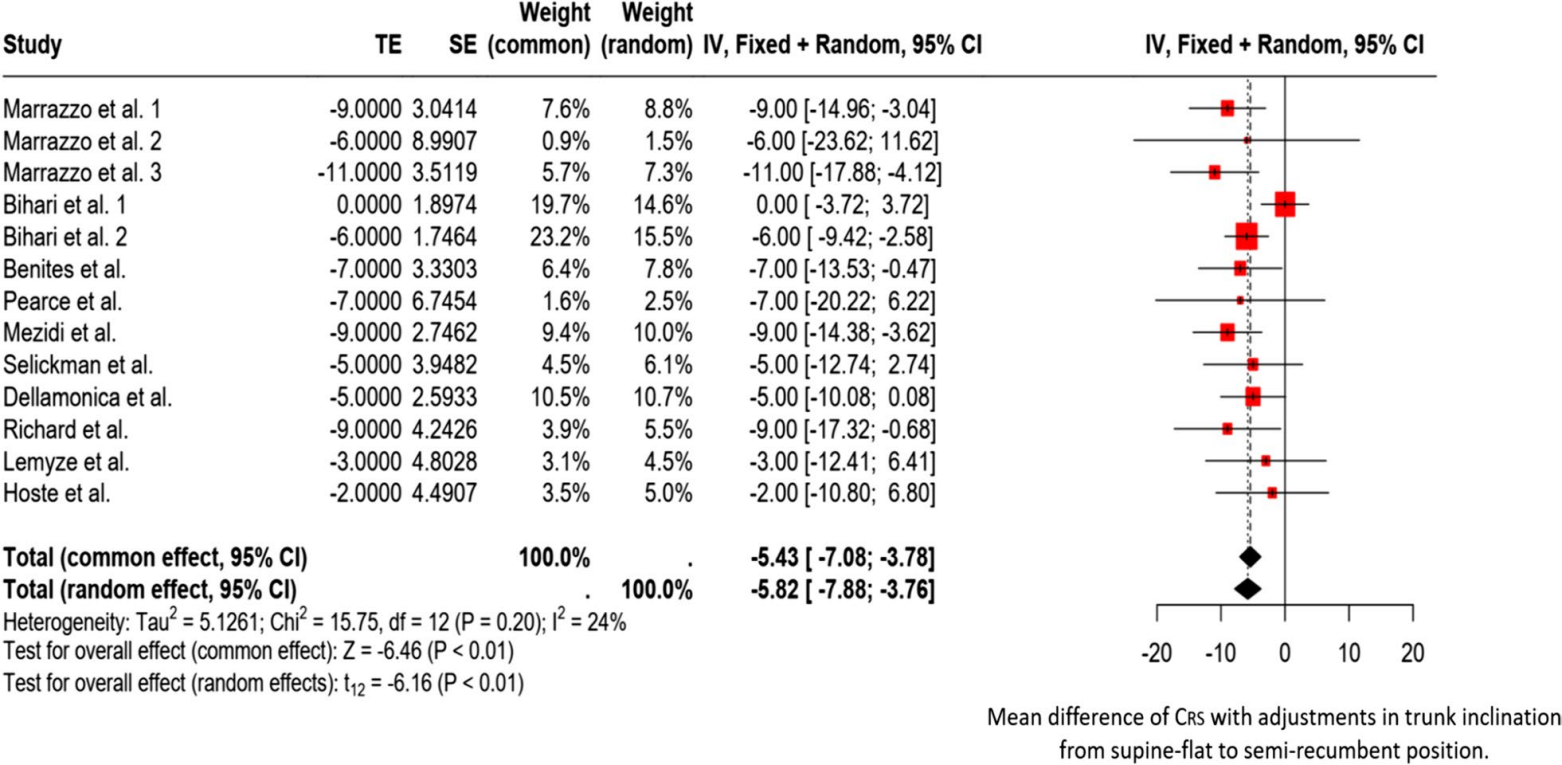
Forest plot. Mean difference of C_RS_ with adjustments in trunk inclination from supine-flat to semi-recumbent position. TE (Treatment Effect) represents the difference in mean compliance of respiratory system between the two positions. SE (Standard Error): Uncertainty associated with the estimation of treatment effects. Weight (common/random): The weight that each study contributes to the calculation of the overall effect under the fixed effects model (common) and the random effects model (random). Confidence Intervals (CI): IV, Fixed + Random; 95% CI, 95% confidence interval for the difference in mean Compliance of respiratory system in both models. Tau^2^, Chi^2^, and I^2^ indicate the degree of heterogeneity among studies. Here, I^2^ = 24% indicates moderate heterogeneity. Tests for Overall Effect: Common Effect: Z =  − 6.46 (*p* < 0.01), indicating a statistically significant combined effect under the fixed effects model. Random Effects: t =  − 6.16 (*p* < 0.01), indicating a statistically significant effect under the random effects model. Bihari et al.1 evaluated non-obese patients with ARDS. Bihari et al. 2 evaluated obese patients with ARDS

Studies have shown that in patients with acute respiratory failure under passive ventilation, transitioning from a semi-recumbent to a supine-flat position in a short assessment period reduces driving and transpulmonary pressure and improves C_RS_ (Fig. 2) [1–3, 6, 8–11, 14, 15]. Except for the study by Hoste et al. [7], these effects have been consistently observed in all evaluated cases of acute respiratory failure [1–3, 6, 8–11, 14, 15]. However, one study found that in non-obese patients with classic ARDS, changes in driving pressure and C_RS_ were not as significant as those in obese patients. In non-obese patients, only chest wall compliance was lower in the supine-flat position than in the semi-recumbent position [14]. It is worth noting that in studies that did not differentiate between obese and non-obese patients, the average body mass index of patients with acute respiratory failure was approximately 30 kg/m^2^ [3, 10, 11]. In addition, changes in trunk inclination will likely require a new PEEP configuration to optimize its values. Without optimization, there is a potential risk of over-distention or collapse of the lungs [11].

2. Impact of trunk inclination adjustment on end-expiratory lung volume and ventilation distribution

Eight studies that evaluated the effect of bed inclination on EELV and ventilation distribution in 158 patients were identified. Of these studies, four focused on patients with classic ARDS (n= 93) [2, 3, 6, 9, 15], three focused on patients with C-ARDS (n= 45) [1, 3, 11], and one evaluated patients with respiratory failure after cardiac surgery (n= 20) [12]. EELV was analysed in 76 patients using the nitrogen washout/washin technique [2, 6, 9]. In addition, 82 patients were examined for regional lung ventilation distribution using electrical impedance tomography [1, 3, 11, 15]. The findings are summarized in Table 1.

**Table 1:** Effects of trunk inclination adjustment on EELV, Ventilation distribution, and Oxygenation

Study	Population (n = 158)	The angle of trunk inclination used	Outcome measures	Conclusions
Richard et al. Intensive Care Med 2006 [6]	ARDS patients (n = 16)	From supine-flat (0°) to semirecumbent head-up (45°) position with lower limbs down at 45°	During immediate vertical positioning, there was a notable difference in EELV between patients who showed an increase in oxygen level (PaO_2_) greater than 40% and those who did not. EELV increased by 500 ± 272 mL in responders and 310 ± 225 mL in non-responders (*p* = 0.286). When patients switched back from the vertical to supine position, responders showed a greater decline in EELV (837 ± 329 mL) than non-responders (320 ± 231 mL, *p* = 0.009)	The higher EELV observed in responders in the vertical position could have resulted from either alveolar recruitment or overdistention
Delllamonica et al. Intensive Care Med 2013 [2]	ARDS patients (n = 40)	From supine-flat (15°) to semirecumbent (45°) position	An increase in EELV/PBW (predicted body weight) was observed exclusively in patients who experienced a PaO_2_/FiO_2_ improvement > 20%. Specifically, in these patients, the EELV/PBW increased from 16 [13–22] to 19 [15–25] ml/Kg (*p* < 0.05). The coefficient of determination between the EELV and PaO_2_/FiO_2_ was R2 = 0.037	Changes in EELV/PBW were observed only in 13 of the 40 patients studied, and no correlation was found between EELV/PBW changes and PaO_2_/FiO_2_ when adjusting for trunk inclination
Spooner et al. Respir Care 2014 [12]	Patients after cardiac surgery (n = 20)	From 0 to 20° of thoracic inclination. Then, from 20° to 30° of thoracic inclination	Switching patients from 0° to 20° increased EELI of 1.054 impedance units (95% CI: 888–1.219, *p* < 0.001). Further elevation to a 30° angle increased EELI by 1.327 impedance units as compared to baseline at 0° (95% CI 1.080–1.573; *p* < .001)	In mechanically ventilated patients after cardiac surgery, increasing the angle of trunk inclination enhances EELI, which suggests improved functional residual capacity
Mezidi et al. Intensive Care Med 2019 [9]	ARDS patients (n = 24)	From supine-flat (0°) to semirecumbent (30°) position	EELV increased from 1203 [994–1412] to 1371 [1137–1606] (*p* = 0.001)	In the supine-flat position as compared to the semi-recumbent position, the decrease in EELV suggests that pleural pressure increases, likely due to the weight of the abdominal contents
Marrazzo et al. Respir Care. 2023 [1]	C-ARDS patients (n = 15)	From supine-flat (0°) to semirecumbent (40°) position	The EELV increased significantly from 123 [− 239– − 54] to 148 [36–300] (*p* < 0.001). In the ventral region, the levels increased from − 52 [− 108– − 13] to 19 [− 16–115] (*p* = 0.003), and in the dorsal region, they increased from − 76 [− 112– − 35] to 98 [30–184] (*p* < 0.001)	Adopting a supine-flat position decreased the global and regional EELV
Pearce et al. Crit Care Explor 2023 [15]	ARDS patients (n = 13)	From supine at 0° to semirecumbent head-up at 35–40°	The dorsal fraction of ventilation was significantly lower in the supine position than in the semi-recumbent position (48.5% vs. 54.5%, *p* = 0.003), indicating an increase in ventral ventilation when the patients were laid flat. The center of ventilation shifted ventrally in the supine position (47.9 vs. 49.5, *p* = 0.005)	The supine position may result in a more favorable distribution of ventilation than the semi-recumbent position, potentially reducing ventral overdistension and improving C_RS_
Benites et al. Intensive Care Med Experimental 2023 [3]	C-ARDS patients (n = 18), ARDS (n = 4)	From supine-flat at 10° to semirecumbent position at 45°	The impedance ratio (ratio between ventral and dorsal ventilation) significantly increased from 0.86 [IQR 0.51–1.33] to 1.27 [IQR 0.83 to 1.78] (*p* < 0.001). Regional tidal volume decreased only in the dorsal left region from 298 [211–403] to 225 [120–288] (*p* = 0.007). The global inhomogeneity index remained stable (*p* = 0.700), and the EELI showed no significant changes in any of the four lung quadrants (ventral and dorsal regions)	The impedance ratio increased, showing a shift in air distribution from dependent to nondependent lung areas. When patients switched to the semi-recumbent position, the EELI levels increased in the dorsal lung regions. This may be attributed to structural changes and decreased superimposed anatomical pressure in the specific lung area
Marrazzo et al. Journal of clinical medicine 2023 [11]	C-ARDS patients (n = 12)	Comparison of PEEP setting between supine-flat position at 0° and semi-recumbent position at 40°	Variations EELV were calculated from changes in end-expiratory lung impedance (EELI) recorded. At the titrated PEEP level, changes in EELV (ΔEELV) over 30 min were − 153 ± 162 mL in the supine-flat vs. + 27 ± 203 mL in the semi-recumbent position (*p* = 0.007) Ventral Region Volume: − 61 ± 94 mL in the supine-flat vs. − 30 ± 127 mL in the semi-recumbent position (*p* = 0.39) Dorsal Region Volume: − 91 ± 80 mL in the supine flat vs. + 57 ± 89 mL in the semi-recumbent position (*p* < 0.001)	Lower PEEP levels were required in the semi-recumbent position than in the supine-flat position to optimize the respiratory mechanics. This is likely due to the increase in EELV in the semi-inclined position, which shifts the pressure–volume curve of the respiratory system towards higher areas. However, at the optimal PEEP level, in the semi-recumbent position, EELV was more stable than in the supine-flat position over the 30 min of observation

EELV: End-expiratory lung volume; PaO2/FIO2: PaO2 over the fraction of inspired oxygen; ARDS: Acute respiratory distress syndrome; C-ARDS: COVID-19-associated acute respiratory distress syndrome; PBW: predicted body weight; PEEP: Positive end-expiratory pressure; CRS: Respiratory system compliance; EELI: End-expiratory lung impedance

The effects of changes in trunk inclination on EELV are highly heterogeneous across studies. The results may show an increase in EELV when moving from a flat supine position to a semi-recumbent one [2, 6, 9]. However, the transition from supine to seated position showed an increase in end-expiratory lung volume (EELV) in patients who experienced an increase in oxygen levels (PaO_2_) greater than 40%. These patients exhibited an EELV increase of 500 ± 272 mL, whereas those who did not show improvement in oxygenation exhibited a minor EELV increase of 310 ± 225 mL (*p*=0.286) [6]. Similarly, Dellamonica et al. observed an increase in the EELV/PBW (predicted body weight) ratio from 16 [13–22] to 19 [15–25] ml/kg (*p* < 0.05) [2] with a change in trunk inclination supine-flat to a sitting position. Specifically, in patients who experienced an improvement in the PaO_2_/FiO_2_ ratio of > 20% when placed in a sitting position compared to a flat supine position, the EELV/PBW ratio increased significantly from 14 (13-15) ml/ kg to 16 (14-20) ml/kg (*p* < 0.05) [2]. In turn, Mezidi et al. observed an increase in EELV from 1203 [994–1412] to 1371 [1137–1606] (*p* = 0.001) with a transition from a supine flat to a semi-recumbent position [9].

Marrazzo et al. observed that transitioning from the supine position (0°) to the semi-recumbent position (40°) resulted in a decrease in ventilation distribution in the ventral (non-dependent) regions and an increase in the dorsal (dependent) regions of the lung. Specifically, ventral ventilation decreased from 57 ± 9% to 53 ± 10% (*p*=0.01), while dorsal ventilation increased from 43 ± 9% to 47 ± 11% (*p*=0.01). These changes in ventilation distribution were associated with a marked decrease in ventral regional compliance, which dropped from 23 ± 9 ml/cmH_2_O in the supine position to 15 ± 6 ml/cmH_2_O in the semi-recumbent position (*p*<0.001), representing an approximate 30% decrease. The reduction in dorsal compliance was less pronounced, decreasing from 17 ± 5 ml/cmH_2_O to 14 ± 5 ml/cmH_2_O (*p*=0.02) [1]. Additionally, Pearce et al. observed that the dorsal fraction of ventilation was significantly lower in the supine position compared to the semi-recumbent position (48.5% vs. 54.5%, *p*=0.003), indicating an increase in ventral ventilation when the patients were laid flat. Furthermore, the center of ventilation shifted ventrally in the supine position (47.9 vs. 49.5, *p*=0.005) [15]. Benites et al. observed that the tidal variation of impedance decreased only in the dorsal left region, from 298 [211–403] to 225 [120–288] (*p*=0.007), when patients were moved from the supine-flat to the semi-recumbent position. The global inhomogeneity index remained stable (*p*=0.700), and EELI showed no significant changes in any of the four lung quadrants (ventral and dorsal regions). However, when patients were placed in the supine-flat position, the impedance ratio (ratio between ventral and dorsal ventilation) significantly increased from 0.86 [IQR 0.51–1.33] to 1.27 [IQR 0.83 to 1.78] (*p*<0.001). This effect was accompanied by an increase in the VTI in the dorsal left region [3]. Finally, Spooner et al. observed that in patients with acute respiratory failure after cardiac surgery, increasing the angle of trunk inclination enhances end-expiratory lung impedance (EELI). Switching patients from 0° to 20° increased EELI by 1.054 impedance units (95% CI: 888–1.219, *p* < 0.001). Further elevation to a 30° angle increased EELI by 1.327 impedance units compared to the baseline at 0° (95% CI: 1.080–1.573, P < 0.001) [12].

3. Effects of trunk inclination adjustment on oxygenation

**Fig. 3:**
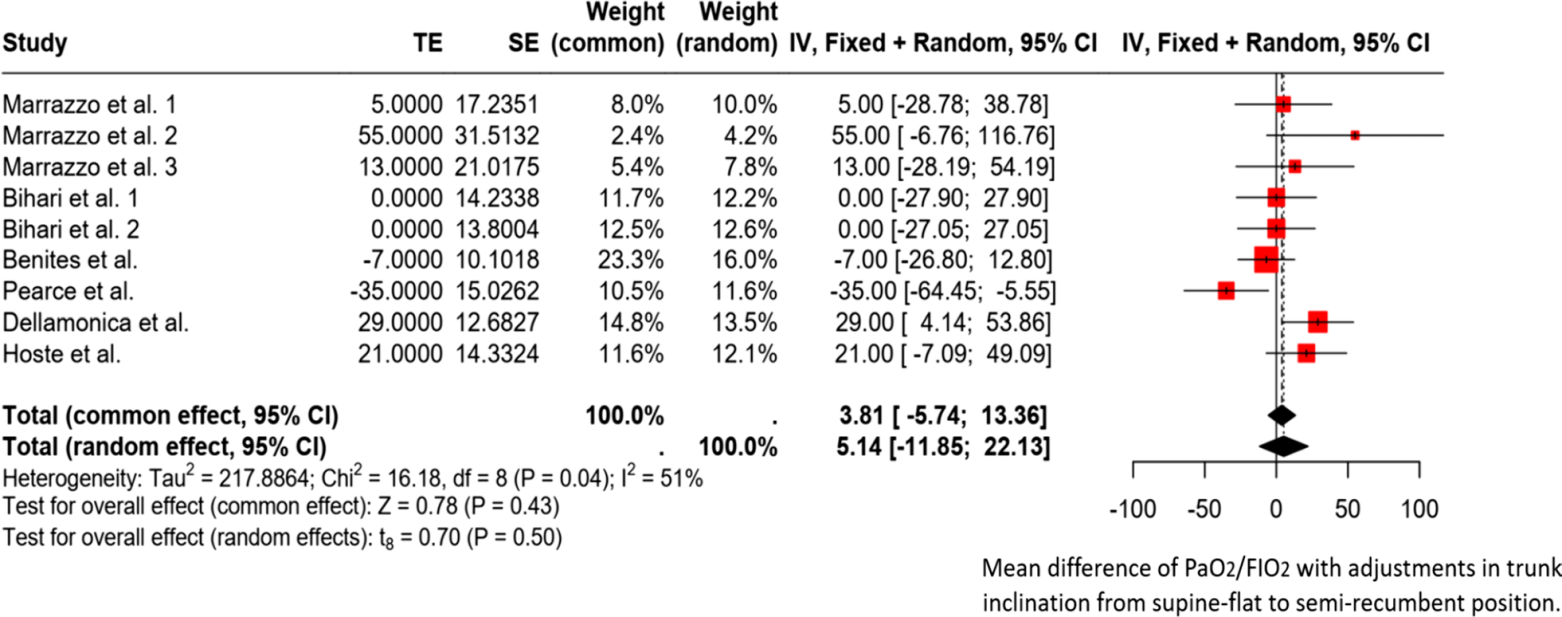
Forest plot. Forest plot. Mean difference of PaO_2_/FIO_2_ with adjustments in trunk inclination from supine-flat to semi-recumbent position. TE (Treatment Effect) represents the difference in mean PaO_2_/FIO_2_ between the two positions. SE (Standard Error): Uncertainty associated with the estimation of treatment effects. Weight (common/random): The weight that each study contributes to the calculation of the overall effect under the fixed effects model (common) and the random effects model (random). Confidence Intervals (CI): IV, Fixed + Random; 95% CI, 95% confidence interval for the difference in mean PaO_2_/FIO_2_ in both models. Tau^2^, Chi^2^, and I^2^ indicate the degree of heterogeneity among studies. Here, I^2^ = 51% indicates moderate heterogeneity. Tests for Overall Effect: Common Effect: Z = 0.78 (*p* < 0.43), indicating a significant combined effect under the fixed-effects model. Random Effects: t = 0.70 (*p* < 0.5), indicating a non-significant effect under the random-effects model

Six studies evaluated the effect of bed inclination on oxygenation in 165 patients. All these studies focused on patients with classic ARDS [2, 3, 6, 14] and C-ARDS [1, 3, 10, 11] (Figure 3).

One study demonstrated a significant improvement in arterial oxygenation following the transition from the supine-flat to semi-recumbent positions. Specifically, 11 of 16 patients diagnosed with ARDS exhibited an average increase in PaO_2_ of 91 ± 31% [6]. Another study revealed a significant overall increase in the PaO_2_/FiO_2_ ratio in 40 patients with ARDS, from 131 [116–180] to 160 [122–210] [2]. However, only 13 of the 40 patients (32%) showed improved oxygenation. In this specific group, the PaO_2_/FiO_2_ ratio increased from 130 (interquartile range: 110–151) mmHg in the supine position to 210 (interquartile range: 175–222) mmHg in the seated position, highlighting the significance of their contribution to the overall study results. Furthermore, statistical analysis revealed no significant correlation between alterations in the normalized EELV to predict body weight and changes in the PaO_2_/FiO_2_ ratio (r^2^ = 0.07).

In obese patients with ARF, there was no significant difference in PaO_2_/FIO_2_ between the supine-flat and semi-recumbent positions [14]. One study revealed that the semi-recumbent position required a lower positive end-expiratory pressure (PEEP) setting than the supine-flat position to prevent alveolar overdistension [11]. Conversely, three subsequent studies assessing the influence of trunk inclination failed to replicate these improvements in oxygenation [1, 3, 10]. The findings are summarized in Additional file 1.

4. Effects of trunk inclination adjustment on PaCO_2_ and Ventilatory efficiency

**Fig. 4:**
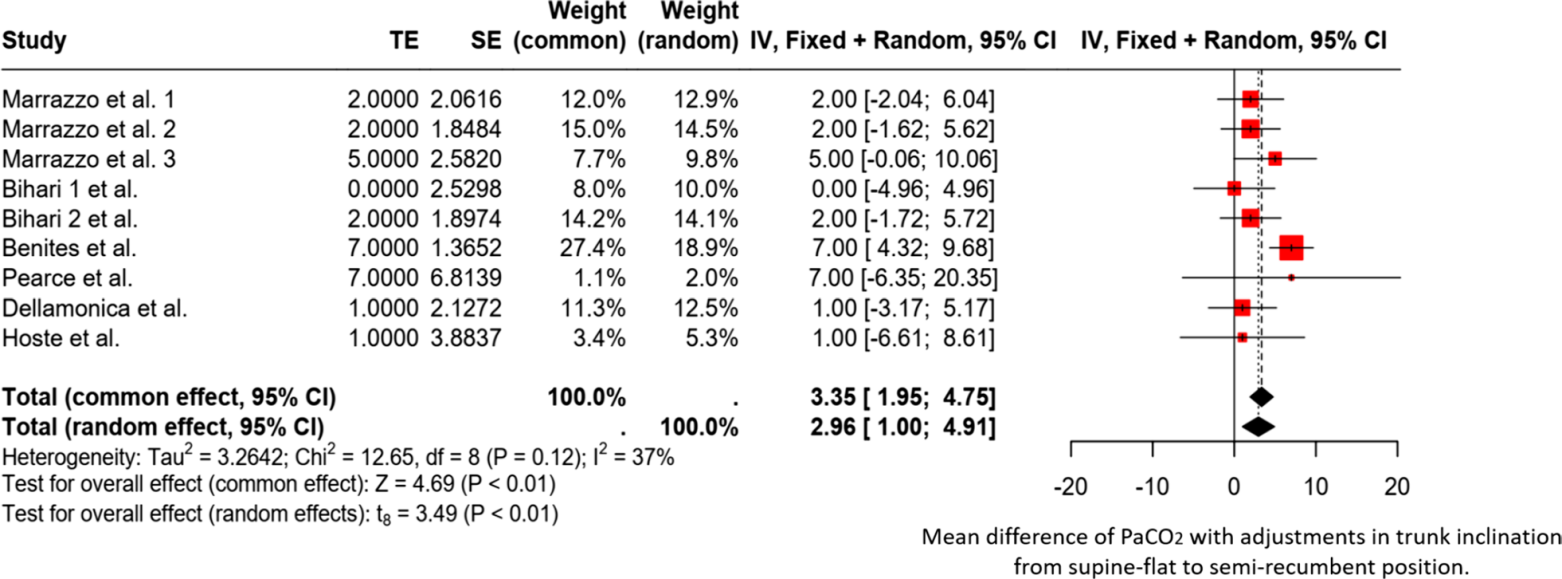
Forest plot. Forest plot. Mean difference of PaCO_2_ with adjustments in trunk inclination from supine-flat to semi-recumbent position. TE (Treatment Effect) represents the difference in mean PaCO_2_ between the two positions. SE (Standard Error): Uncertainty associated with the estimation of treatment effects. Weights: Weight (common/random): The weight that each study contributes to the calculation of the overall effect under the fixed effects model (common) and the random effects model (random). Confidence Intervals (CI): IV, Fixed + Random; 95% CI, 95% confidence interval for the difference in mean PaCO_2_ in both models. Tau^2^, Chi^2^, and I^2^ indicate the degree of heterogeneity among studies. Here, I^2^ = 37% indicates moderate heterogeneity. Tests for Overall Effect: Common Effect: Z = 4.69 (*p* < 0.01), indicating a significant combined effect under the fixed-effects model. Random Effects: t = 3.49 (*p* < 0.01), indicating a significant effect under the random-effects model

Eight studies evaluated the effects of bed inclination on PaCO_2_ and ventilatory efficiency in 149 patients [1–3, 7, 10, 11, 14, 15]. All of these studies focused on patients with ARDS (n=115) [2, 3, 14, 15] and C-ARDS (n=65) [1, 3, 10, 11]. Eight studies [1–3, 7, 10, 11, 14, 15] assessed PaCO_2_ levels (n=149), three studies (n=57) evaluated the ventilatory ratio [1, 3, 11], and three studies examined the dead space (n=57) [1, 3, 11]. However, only one study provided a detailed methodology for assessing ventilatory efficiency/inefficiency using volumetric capnography [3]. A forest plot (Figure 4) summarizes the effect of trunk inclination on PaCO_2_. The accompanying supplementary file summarizes all studies.

The forest plot shows that changing from a supine to a semi-recumbent position is associated with a significant increase in PaCO_2_. The combined effects, represented by the diamond, highlight this increase. The heterogeneity is moderate (I^2^ = 37%), suggesting that although there is some variability among the studies, the results are mostly consistent. In the subgroup analysis, it is evident that in patients with C-ARDS, the increase in PaCO_2_ with the change in trunk inclination from supine-flat to semi-recumbent is significantly greater than that in patients with classic ARDS (Additional file 1).

## Discussion

After analysing 13 studies, this scoping review identified a diverse population of patients with various respiratory disorders and post-surgical conditions. There was considerable variability in patient oxygenation levels at baseline, reflecting a broad spectrum in the severity of their conditions. Furthermore, we found significant variability in the outcomes evaluated, demonstrating substantial differences in clinical profiles and treatment responses within this heterogeneous population.

In many studies focusing on patients with acute respiratory failure, transitioning from a semi-recumbent to a supine-flat position markedly improved respiratory mechanics. This improvement was evidenced by a notable increase in the C_RS_ and a decrease in airway driving pressure. This positional adjustment has been associated with substantial reductions in PaCO_2_ and enhancements in ventilatory efficiency, particularly in patients with C-ARDS. In contrast, such benefits appear to be less evident in classic ARDS patients. A universally consistent observation across these studies was the absence of any improvement in oxygenation levels. An in-depth analysis of these physiological effects is provided in the subsequent sections.

### Trunk inclination adjustment and its effects on respiratory mechanics

Consistently, it has been shown in patients with classic ARDS and C-ARDS, who are under mechanical ventilation without activation of the respiratory muscles, that changing from a semi-recumbent to a supine-flat position decreases airway driving pressure and increases C_RS_ [1, 9, 10, 14]. This effect can be explained by the fact that adjusting the bed's inclination angle towards a more supine-flat position allows positioning of the lungs in a better part of their pressure-volume curves, which varies according to the set level of PEEP [1, 11].

Marrazo and colleagues evaluated the optimal PEEP level in the supine-flat and semi-recumbent positions [11]. They aimed to find a balance between overdistension and lung collapse in each posture using electrical impedance tomography (EIT). This tool helps establish the optimal PEEP level chosen based on a balance between the proportion of hyperinflated and collapsed pixels. It is a valuable bedside approach to optimize PEEP; nonetheless, it presents several limitations. These authors observed that the optimal PEEP differed by approximately 5 cmH_2_O with changes in trunk position. Thus, the interplay between changes in respiratory mechanics and PEEP adjustment, along with changes in trunk inclination angles, should be considered. A PEEP level deemed optimal in the supine-flat position can lead to overdistension if this setting is not modified when adopting a semi-recumbent position. Conversely, optimally adjusting the PEEP for the semi-recumbent position can relieve overdistension in non-dependent areas of the lung and may cause dependent lung collapse when the patient is placed in a supine-flat position. However, considering that in that study the PEEP was individualized to optimize both collapse and overdistension in both positions, the finding of a significantly lower driving pressure in the supine-flat position is intriguing. We hypothesize that the improved respiratory system mechanics in this position could be due to at least two mechanisms. First, it is possible that there is less lung mechanic heterogeneity in the supine-flat position, because of the smaller gradient of pleural pressures in this position than in the semi-recumbent position. This effect can be attributed to changes in the vertical height of the lung relative to the body's position, where the vertical height of the lung is minimized in the supine-flat position due to perfect alignment with the ventrodorsal axis. From this position, the lung vertical height progressively increases until it reaches the upright (90°) position, spanning the entire craniocaudal axis. Therefore, in the semi-recumbent position, the lung extends over a greater vertical distance, influencing the pleural pressure gradient, amplifying the superimposed lung weight, and favouring dorsal alveolar collapse and atelectasis. Hence, at a certain PEEP level, a change in trunk inclination towards a semi-recumbent position is likely to overdistend already open alveoli in non-dependent regions, which can potentially increase lung stress [1, 9–11]. However, if the PEEP chosen in the semi-recumbent position is low, shifting to a flat supine position may cause a collapse in the dependent lung areas. This finding underscores the importance of carefully setting the PEEP level to the patient's specific posture to balance the ventilation properly. Second, it is possible that chest wall compliance decreases in the semi-recumbent position due to compression of the abdominal contents caused by the thighs, especially in obese patients. In two separate studies, Marrazzo et al. reported increases of 64% and 122% in chest wall compliance and 15% and 21% in lung compliance, respectively, with the change to the supine-flat position [1, 10]. This could explain the higher PEEP levels set during the supine-flat position by Marrazzo et al. [11]. It is important to recognize that an increase in intra-abdominal pressure can cause deformations in the thoracic cage and increase the elastic recoil pressure of the lungs and chest wall, thereby affecting the elastance of the respiratory system and inducing lung collapse in dependent areas at the end of expiration [16, 17]. Research in animal models has shown that 20–60% of the increased pressure in the abdomen is transmitted to the thoracic compartment [18]. This is relevant because changing from a supine-flat to a semi-recumbent position can increase intra-abdominal pressure [19–22]. Therefore, there are solid physiological bases to consider that these adjustments in trunk inclination to a semi-recumbent position and changes in intra-abdominal pressure significantly contribute to the mechanical alterations observed at the respiratory system level [23, 24]. In addition, when the airway or intra-abdominal pressure significantly increases, the diaphragm balances the energy transfer between the two compartments [18], leading to evident deformations and displacements.

### Trunk inclination adjustment and its effects on end-expiratory lung volume and ventilation distribution

In patients with ARDS, there is a dramatic decrease in functional residual capacity due to gravitational gradients in the lungs, causing alveolar collapse in dependent areas accompanied by impaired oxygenation [25]. Two studies have shown that a semi-recumbent posture can increase the EELV in patients with classic ARDS, but only in some patients [2, 6].

Adopting a supine-flat position may result in a notable decrease in the end-expiratory lung impedance (EELI) in the ventral region in some patients. This effect, characterized by its rapid onset and reversibility, is primarily attributed to enhanced respiratory mechanics stemming from a reduction in ventral alveolar hyperinflation and a corresponding increase in regional lung compliance [1, 15]. This phenomenon is likely due to the pressure‒volume curves shifting towards the middle zone, which contributes to the overall improvement in lung compliance [11]. This observation contrasts with the findings of Benites et al. [3], where neither the ventral nor dorsal regions displayed substantial EELI changes between the 45° and 10° positions. The variation in EELI among ARDS patients in response to trunk inclination could be linked to the varying severity of ARDS among the study participants, which ranged from mild to severe. The physiological response to changes in trunk position is primarily determined by recruitable lung tissue. For example, in moderate or severe ARDS, in which lung recruitment can be limited, an upright posture leading to increased transpulmonary pressure may result in minimal or no lung recruitment. This predominantly causes overdistension, particularly in patients with more severe forms of the syndrome [1, 9, 10]. In turn, numerous studies have expressed the EELI and tidal variation in impedance (VTI) in different formats (as percentages or indexed to VT). Interpreting such findings remains debatable, and a consensus still needs to be reached.

On the other hand, a reduction in the impedance ratio has been highlighted, indicating an enhancement in the ventilation distribution in the dorsal lung regions with adjustment of the trunk to a supine-flat position [3]. Upon further analysis of the ventilation distribution, which was mainly segmented by distinct lung areas, an increase in the VTI in the dorsal left region was noted. This increase suggests that the overlying of the heart on the lung tissue [15, 26] marks this shift in ventilation as a significant contributor to the observed physiological effect. In contrast, Pearce et al. observed that the dorsal fraction of ventilation was significantly lower in the supine-flat than in the semi-recumbent position [14] and Marrazo et al. observed that a greater distribution of ventilation in the ventral areas of the lung was associated with a gain in compliance measured by EIT [1]. Explaining these results regarding ventilation distribution poses challenges owing to methodological disparities across studies [1, 3, 14]. First, the use of pressure-controlled ventilation [3], in contrast to volume-controlled ventilation [1, 10, 14], increased VT in response to increased C_RS_. In this scenario, an increase in VT could have induced tidal recruitment in the dorsal regions with variable effects on ventral overdistention. Conversely, under constant VT conditions with improved C_RS_, a reduction in ventral overdistension can occur. Second, an extended timeframe per position (60 min [3] vs. 15 min [1] vs. 30 min [14]) could demonstrate evolutive ventilatory changes that could have been overlooked during the 15-minute assessment.

### Effects of trunk inclination adjustment on oxygenation

Shifting from a supine-flat to a semi-recumbent position can increase oxygenation in some ARDS patients due to multiple factors. This change primarily aids in reopening the collapsed alveoli, leading to an increase in EELV [2, 6] via an increase in transpulmonary pressure [1, 11, 14]. However, when patients are initially set with optimal PEEP in a supine-flat position and then moved to a semi-recumbent position, lung stress may increase, indicating potential overdistension [11]. This situation highlights the necessity of re-evaluating the PEEP settings to maintain optimal lung support when changing the patient's posture. Marrazo et al. observed a significant improvement in oxygenation despite using a lower PEEP setting in the semi-recumbent position [11]. Accordingly, careful adjustment of the PEEP level is required to optimize gas exchange and prevent lung overdistension during trunk inclination.

### Effects of trunk inclination adjustment on PaCO_2_ and ventilatory efficiency

Available data indicate that shifting from a semi-recumbent to a supine-flat position decreases PaCO_2_ [1–3, 10, 14]. Additionally, when assessments were conducted after 30 minutes at the optimal PEEP level using EIT in both positions, similar levels of PaCO_2_ were observed. This observation further supports the hypothesis that alveolar collapse/overdistension may significantly impact suboptimal CO_2_ clearance when PEEP is inadequately adjusted in the semi-recumbent position [11]. In contrast, Dellamonica et al. reported no significant changes in PaCO_2_ levels related to shifts in thoracic inclination in a group of 40 ARDS patients [2]. It is crucial to not overlook the influence of various ARDS etiologies, such as COVID-19, compared to typical ARDS, on these results.

In addition, when the trunk is inclined from the semi-recumbent to supine-flat position an increase in exhaled CO_2_ per minute, is observed. Accordingly, a reduction in Bohr dead-space is generated, suggesting less overdistension [3]. This finding is reinforced by a marked decrease in the slope of phase III (SnIII) in the capnogram when patients are in a supine-flat position, indicating more efficient CO_2_ exchange. However, whether a change in trunk position can improve pulmonary circulatory efficiency or reduce the shunt effect remains unclear [3]. Another essential consideration is the impact of evaluation time on outcomes. No significant changes in the parameters captured by volumetric capnography were observed between the 15- and 60-minute intervals in the supine-flat position. This evidence suggests that modifications in trunk inclination induce rapid alterations in CO_2_ clearance and that these alterations are sustained with minimal fluctuations over a 60-minute observational period [3].

Concurrent physiological conditions can impact alveolar ventilation. Alveolar overdistension is one of the most significant mechanisms that may disrupt CO_2_ exchange. Consistently, transpulmonary pressure and driving pressure are elevated in the semi-recumbent position. This could lead to further inflation of already open alveoli, particularly in the ventral regions of the lung. Furthermore, transitioning to a semi-recumbent position can cause the abdominal contents to exert pressure on the diaphragm, reducing the space available for lung expansion. This could further complicate respiratory mechanics and impair alveolar ventilation and gas exchange [22].

**Fig. 5:**
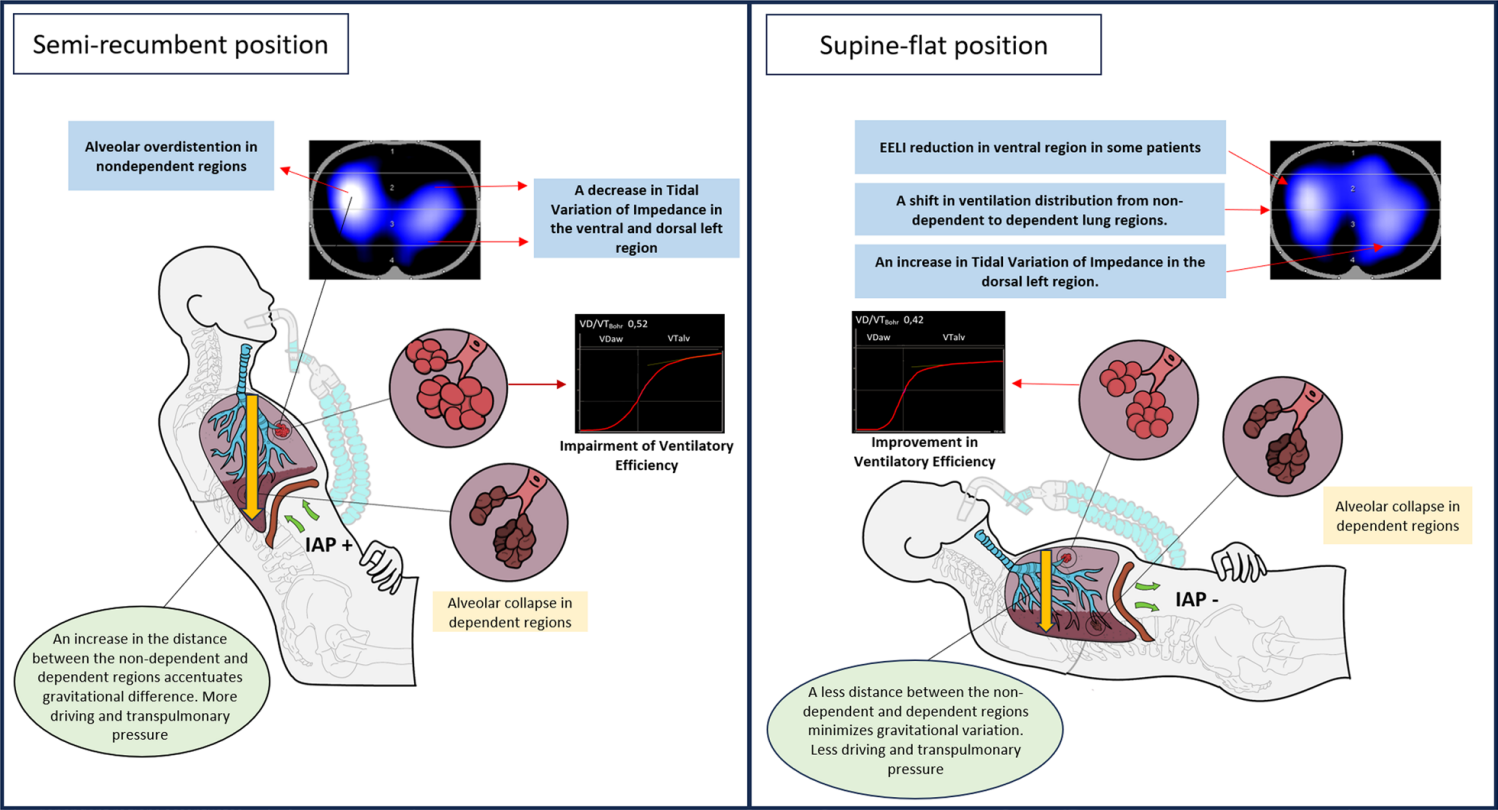
Summarizes the main physiological effects of trunk inclination in patients with C-ARDS

The following scheme summarizes the most remarkable effects of changes in trunk inclination in patients with acute respiratory failure (Figure 5).

Schematic representation of a patient with acute respiratory failure connected to mechanical ventilation showing two different trunk inclinations and respiratory effects. Monitoring is performed using electrical impedance tomography, which allows the assessment of changes in the distribution of inspired air. 'EELI' stands for end-expiratory lung impedance. A graphical interface for volumetric capnography was also included to facilitate visualization of changes in exhaled CO_2_. 'IAP+' indicates an increase in intra-abdominal pressure; 'IAP-' denotes a reduction in intra-abdominal pressure.

More needs to be known regarding the mechanisms underlying these physiological effects. For instance, it is unclear how changes in trunk inclination affect regional lung perfusion and transpulmonary pressure in dependent and nondependent regions. Additionally, how body inclination affects intra-abdominal pressure and energy transfer to the thoracic cage with trunk inclination remains unclear. Since hemodynamic changes can be influenced by increases in intra-abdominal pressure and reduced chest wall compliance [23, 26, 27], critical conditions, such as hypovolemia or right ventricular dysfunction, are expected to affect the hemodynamic stability of these patients with changes in trunk inclination. Consequently, these changes may influence the ventilation-perfusion ratio. Therefore, studies that holistically evaluate these physiological changes and integrate the assessment of systems beyond the lungs are needed.

### Final comments and clinical massage

It is important to note that the degree of inclination of the bed should not only be recorded in clinical practice but also be required in research studies that perform physiological assessments of patients with respiratory failure. This is because the results obtained can be very different depending on the angle of inclination of the bed for each participant and can generate erroneous conclusions. It is essential to record the angle of the bed and conduct a functional respiratory assessment to objectively evaluate the clinical repercussions of this manoeuvre. The intrinsic variability of ARDS underscores the need for mechanical ventilation strategies tailored to the individual characteristics of each patient. This personalized approach, grounded in the principles of precision medicine, is gaining recognition and appreciation in clinical practice.

### Limitations

Our research strategy was narrowly tailored to include only those studies published in traditional academic venues. We did not contact the authors for individual patient data but relied solely on published literature for our data extraction. This review covers a variety of populations with acute respiratory failure without focusing on a specific etiology. The heterogeneous reporting of outcomes across individual studies made data extraction difficult, limiting our analysis to a descriptive format. While we addressed this research question, the current evidence remains inconclusive, leaving many unresolved hypotheses for future studies. Likewise, these short assessment periods did not allow for evaluating adverse events, such as ventilator-associated pneumonia.

## Conclusions

In patients with acute respiratory failure, transitioning from a supine to a semi-recumbent position leads to decreased respiratory system compliance and increased airway driving pressure. Additionally, C-ARDS patients experienced an improvement in ventilatory efficiency, which resulted in lower PaCO_2_ levels. Improvements in oxygenation were observed in a few patients and only in those who exhibited an increase in EELV upon moving to a semi-recumbent position. Therefore, the trunk inclination angle must be accurately reported in patients with respiratory failure under mechanical ventilation.

### Supplementary Information

Additional file 1.

Additional file 2.

## Data Availability

The data sets used and analyzed during the current study are available from the corresponding author on reasonable request.

## Abbreviations

PaCO_2_: Pressure of carbon dioxide
EELV: End-expiratory lung volume
PaO_2_/FIO_2_: PaO_2_ over the fraction of inspired oxygen
ARDS: Acute respiratory distress syndrome
C-ARDS: COVID-19-associated acute respiratory distress syndrome
PEEP: Positive end-expiratory pressure
C_RS_: Respiratory system compliance
EELI: End-expiratory lung impedance
EIT: Electrical impedance tomography
