# Supplementary material for: Physiological and clinical effects of trunk inclination adjustment in patients with respiratory failure: a scoping review and narrative synthesis
Source: Crit Care. 2024 Jul 9;28:228. doi: 10.1186/s13054-024-05010-1 (PMC11232125; doi:10.1186/s13054-024-05010-1)
Supplement: Supplementary file 1 — Additional file 1. [file 13054_2024_5010_MOESM1_ESM.docx]

**Physiological and clinical effects of trunk inclination adjustment in patients with respiratory failure. A Scoping review and narrative synthesis**

**Additional file 1**

Table of Contents

[eTable 1. Overview expert panel 2](#_Toc169869569)

[METHODS 2](#_Toc169869570)

[eTable 2. Preferred Reporting Items for Systematic reviews and Meta-Analyses extension for Scoping Reviews (PRISMA-ScR) Checklist 6](#_Toc169869571)

[e-Table 3. Change in thoracic inclination and its effects on respiratory mechanics 7](#_Toc169869572)

[e-Table 4. Change in thoracic inclination and its effects on oxygenation 10](#_Toc169869573)

[eTable 5. Effects of trunk inclination on PaCO2 and Ventilatory inefficiency 12](#_Toc169869574)

[Forest Plot: PaCO2 14](#_Toc169869575)

[Forest Plot: Compliance of respiratory system (C_RS_) 15](#_Toc169869576)

[Forest Plot: PaO2/FIO2 16](#_Toc169869577)

[Registered Search Strategy 17](#_Toc169869578)

## eTable 1. Overview expert panel

| **Name** | **Medical background** | **Department** | **Research lines** |
| --- | --- | --- | --- |
| *Martín Benites* | Master in Epidemiology. Doctoral student in Medical Science | Department of Critical Care, Clínica Las Condes. Universidad Finis Terrae. Pontificia Universidad Católica de Chile | ARDS, Mechanical ventilation |
| *Marcelo Zapata* | Master in Epidemiology. Systematic Review methodologist | Department of Medicine, Division of Critical Care. McMaster University, Hamilton. Canada | ARDS |
| *Eduardo Costa* | Ph.D. Intensivist. | Laboratório de Pneumologia LIM-09, Disciplina de Pneumologia, Heart Institute (Incor), Hospital das Clínicas da Faculdade de Medicina da Universidade de Sao Paulo, São Paulo, Brazil | ARDS, Mechanical ventilation |
| *Alejandro Bruhn* | Ph.D. Intensivist. | Department of Critical Care. Pontificia Universidad Católica de Chile | ARDS, Mechanical ventilation |
| *Guillermo Bugedo* | Profesor. Intensivist | Department of Critical Care. Pontificia Universidad Católica de Chile | ARDS, Mechanical ventilation |
| *Jaime Retamal* | PhD. Internal Medicine,  Intensive Care | Department of Critical Care. Pontificia Universidad Católica de Chile | ARDS, Mechanical ventilation |

## METHODS

This scoping review followed the PRISMA-ScR guidelines and unfolded using a streamlined five-stage methodology. It begins with establishing a research question, advances with identifying and selecting pertinent studies, proceeds to systematically charting data, and culminates in the comprehensive summarization and reporting of results. This approach is grounded in the framework developed by Arksey and O’Malley and further refined by Levac et al., ensuring a thorough and precise synthesis of the available literature.

- Tricco AC, Lillie E, Zarin W, O’Brien KK, Colquhoun H, Levac D, et al (2018) PRISMA extension for scoping reviews (PRISMA-ScR): checklist and explanation. Ann Intern Med 169:467–473. <https://doi.org/10.7326/M18-0850>
- Arksey H, O’Malley L (2005) Scoping studies: towards a methodological framework. Int J Soc Res Methodol 8:19–32. <https://doi.org/10.1080/1364557032000119616>
- Levac D, Colquhoun H, O’Brien KK (2010) Scoping studies: advanc‑ ing the methodology. Implement Sci 5:69.

https://doi.org/10.1186/ 1748-5908-5-69

- **Stage 1: identifying a research question.**

What are the clinical and physiological impacts of adjusting the trunk from a supine-flat to a semi-recumbent position on the respiratory function in patients with acute respiratory failure?

- **Stage 2: identifying relevant studies**

Extensive searches were conducted across databases, such as PubMed, Cochrane, and Scopus, from 2003 to March 2024. For an in-depth look at the search methodology used, please see the following section, which includes the sample search strategy.

- **Stage 3: study selection**

Studies were selected based on the publication year, title, and abstract. Two independent reviewers conducted this initial screening, and disagreements were resolved through mutual discussion or the involvement of a third reviewer. We also included a risk of bias assessment to enhance discussions about study quality and provide directions for future research. Two independent reviewers performed this assessment similarly, with a third reviewer available to resolve discrepancies.

- **Stage 4: eligibility**

The study criteria focused on adults aged 18 years and over with respiratory failure, invasively ventilated in an ICU, and with less than seven days of mechanical ventilation. Eligibility required studies to involve adjustments to trunk inclination in the supine position, specifically including semi-recumbent, reverse Trendelenburg, or supine-flat positions. Suitable studies have employed experimental models or a repeated-measures design, allowing patients to serve as their own controls, and investigated measures of respiratory mechanics, ventilatory efficiency, oxygenation parameters, end-expiratory lung volume, and ventilation distribution, with assessments potentially using electrical impedance tomography. Inclusion was limited to studies on human participants published in any language between 2003 and 2024.

Conversely, the exclusion criteria were studies without statistical lung function comparisons across different positions, patients undergoing surgery in operating rooms, patients on venovenous ECMO, instances where patients were breathing spontaneously during mechanical ventilation, and studies exclusively focusing on the Trendelenburg position. Additionally, conference abstracts, unpublished materials, case reports, gray literature, observational studies, and systematic or narrative reviews were not considered.

- **Stage 5: charting the data**

Initially, we screened titles and abstracts to remove duplicate entries. Each investigator then performed a more detailed evaluation to shortlist relevant records. In a subsequent in-person meeting, the investigators reviewed the remaining articles, which were included in the scoping review.

The extracted data encompassed publication details, such as author and publication date, and study-specific details, such as the research design and assessments. Given the broad interpretation of 'trunk inclination' in patients with respiratory failure, our search was expanded using backward citation tracking of reference lists of the selected articles.

Key information for each selected study was methodically and independently extracted from the included studies, focusing on the following: 1. Study details; 2. Population characteristics; 3. Design/Article type; 4. Study setting; 5. Trunk inclination angles applied; 6. Outcome measures; 7. Conclusions. Data extraction was conducted independently and redundantly by two reviewers, and any discrepancies were resolved either through discussion or, if required, by consulting a third party for final arbitration.

- **Stage 6: collating, summarizing, and reporting the results**

This study systematically summarized the impact of trunk inclination on various outcomes and organized the data into tables for clarity. As detailed in this file, the authors compiled the results from selected studies, which were subsequently reviewed by an expert committee specializing in respiratory failure and mechanical ventilation. The primary outcomes from each study were categorized into four main areas: Respiratory Mechanics, End-Expiratory Lung Volume or Ventilation Distribution, Oxygenation, and PaCO2 alongside ventilator efficiency. These outcomes were individually analyzed and presented in their respective results tables for detailed examination.

## eTable 2. Preferred Reporting Items for Systematic reviews and Meta-Analyses extension for Scoping Reviews (PRISMA-ScR) Checklist

| **SECTION** | **ITEM** | **PRISMA-ScR CHECKLIST ITEM** | **REPORTED ON PAGE #** |
| --- | --- | --- | --- |
| **TITLE** | | | |
| Title | 1 | Identify the report as a scoping review. | 1 |
| **ABSTRACT** | | | |
| Structured summary | 2 | Provide a structured summary that includes (as applicable): background, objectives, eligibility criteria, sources of evidence, charting methods, results, and conclusions that relate to the review questions and objectives. | 1 |
| **INTRODUCTION** | | | |
| Rationale | 3 | Describe the rationale for the review in the context of what is already known. Explain why the review questions/objectives lend themselves to a scoping review approach. | 4 |
| Objectives | 4 | Provide an explicit statement of the questions and objectives being addressed with reference to their key elements (e.g., population or participants, concepts, and context) or other relevant key elements used to conceptualize the review questions and/or objectives. | 4 |
| **METHODS** | | | |
| Protocol and registration | 5 | Indicate whether a review protocol exists; state if and where it can be accessed (e.g., a Web address); and if available, provide registration information, including the registration number. | 5 |
| Eligibility criteria | 6 | Specify characteristics of the sources of evidence used as eligibility criteria (e.g., years considered, language, and publication status), and provide a rationale. | 5,6 |
| Information sources* | 7 | Describe all information sources in the search (e.g., databases with dates of coverage and contact with authors to identify additional sources), as well as the date the most recent search was executed. | 6,7 |
| Search | 8 | Present the full electronic search strategy for at least 1 database, including any limits used, such that it could be repeated. | Supplementary materials |
| Selection of sources of evidence† | 9 | State the process for selecting sources of evidence (i.e., screening and eligibility) included in the scoping review. | 6-7 |
| Data charting process‡ | 10 | Describe the methods of charting data from the included sources of evidence (e.g., calibrated forms or forms that have been tested by the team before their use, and whether data charting was done independently or in duplicate) and any processes for obtaining and confirming data from investigators. | 7 |
| Data items | 11 | List and define all variables for which data were sought and any assumptions and simplifications made. | Supplementary materials |
| Critical appraisal of individual sources of evidence§ | 12 | If done, provide a rationale for conducting a critical appraisal of included sources of evidence; describe the methods used and how this information was used in any data synthesis (if appropriate). | 7-8 |
| Synthesis of results | 13 | Describe the methods of handling and summarizing the data that were charted. | 7-8 |
| **RESULTS** | | | |
| Selection of sources of evidence | 14 | Give numbers of sources of evidence screened, assessed for eligibility, and included in the review, with reasons for exclusions at each stage, ideally using a flow diagram. | 9, 10 |
| Characteristics of sources of evidence | 15 | For each source of evidence, present characteristics for which data were charted and provide the citations. | 9, 10 |
| Critical appraisal within sources of evidence | 16 | If done, present data on critical appraisal of included sources of evidence (see item 12). | 9 |
| Results of individual sources of evidence | 17 | For each included source of evidence, present the relevant data that were charted that relate to the review questions and objectives. | 11 - 20 |
| Synthesis of results | 18 | Summarize and/or present the charting results as they relate to the review questions and objectives. | 11 - 20 |
| **DISCUSSION** | | | |
| Summary of evidence | 19 | Summarize the main results (including an overview of concepts, themes, and types of evidence available), link to the review questions and objectives, and consider the relevance to key groups. | 20 - 29 |
| Limitations | 20 | Discuss the limitations of the scoping review process. | 29 |
| Conclusions | 21 | Provide a general interpretation of the results with respect to the review questions and objectives, as well as potential implications and/or next steps. | 30 |
| **FUNDING** | | | |
| Funding | 22 | Describe sources of funding for the included sources of evidence, as well as sources of funding for the scoping review. Describe the role of the funders of the scoping review. | Click here to enter text. |

## e-Table 3. Change in thoracic inclination and its effects on respiratory mechanics

| **Study** | **Population**  **(n= 254)** | **The angle of trunk inclination used** | **Outcome measures** | **Conclusions** |
| --- | --- | --- | --- | --- |
| Hoste et al.  J Intensive Care Med 2005 (7) | ARDS patients  (n=18) | From supine-flat to semirecumbent (45°) position | No significant change in C_RS_ (from 35 to 38 ml/cmH_2_O) or driving pressure was observed during the 12-hour evaluation. | Changing the bed angle from supine-flat to upright did not significantly affect the C_RS_ or driving pressure. |
| Richard et al. Intensive Care Med 2006 (6) | ARDS patients (n=16) | From supine-flat (0°) to semirecumbent head-up (45°) position with lower limbs down at 45°. | The plateau pressure increased from 29 ±7 to 32 ±9 cmH_2_O; p = 0.043). C_RS_ decreased from 40 ±15 to 31 ±9 mL/cmH_2_O (p < 0.0076) in 11 patients with improved oxygenation. C_RS_ decreased from 47 ±23 to 33 ±10 mL/cmH_2_O (p = 0.23) in 5 patients who did not improve oxygenation. | In ARDS patients, adjusting the bed angle from 0° to 45° increased plateau pressure and decreased C_RS_. |
| Delllamonica et al. Intensive Care Med 2013 (2) | ARDS patients  (n=40) | From supine-flat (15°) to semirecumbent (45°) position | C_RS_ decreased from 36 [27-45] to 31 [25-40] mL/cmH_2_O. Pulmonary strain decreased from the supine-flat to semi-recumbent position (0.38 (0.27; 0.45) vs. 0.31 [0.24; 0.40] (p = 0.006)). | Shifting from a supine-flat to a semi-recumbent position decreased the C_RS_. |
| Lemyze et al. Crit Care Med. 2013 (13) | Obese Patients (n=16) | From supine-flat (0°) to seated position (70°) | Reduced expiratory flow limitation (from 60% to 0%) and autoPEEP (from 10 cmH2O to 1 cmH2O). Additionally, this positional change led to a decrease in the plateau pressure from 22 cm H2O to 16 cm H2O. All the changes were statistically significant (p < 0.001). | In obese patients, expiratory flow limitation is common in the supine position, which can lead to auto-peep. Changing to a seated position with optimal PEEP settings can reverse EFL and reduce plateau pressures, thus improving respiratory mechanics in this patient group. |
| Mezidi et al. Intensive Care Med 2019 (9) | ARDS patients  (n= 24) | From supine-flat (0°) to semirecumbent (30°) position | C_RS_ decreased from 48 [42–56] to 38 [33–45] mL/cmH_2_O. Lung compliance decreased from 67 [56–77] to 56 [45–71] mL/cmH_2_O. Chest wall compliance decreased from 143 [111–167] to 111 [91 – 143] mL/cmH_2_O. The gastric pressure increased from 12 cmH_2_O [10–15] to 14 [11–17] (p = 0.001). | Shifting from a supine-flat to semi-recumbent position decreased C_RS_, lung compliance, and chest wall compliance. The gastric pressure increased at 30° compared to that in the supine-flat position. |
| Marrazzo et al. Am J Respir Crit Care Med 2022 (10) | C-ARDS patients  (n= 20) | From supine-flat (0°) to semirecumbent (40°) position | C_RS_ decreased from 38 [33–48] to 29 [24-35] ml/cmH_2_O. The lung compliance decreased from 46 [40–62] to 38 [30–46] mL/cmH_2_O. Chest wall compliance decreased from 215 [175–300] to 131 [101–170] mL/cmH_2_O. The driving pressure increased from 10 to 13 cmH_2_O. | The change in body position from supine-flat to a semi-recumbent position at 40° deteriorated the lung and chest wall mechanics, leading to increased driving pressures. |
| Selickman et al. Crit Care Med 2022 (8) | ARDS patients  (n= 17) | Semi-recumbent angle, with the head of the bed elevated to 30° and the lower extremities parallel to the floor vs. the lower limb tilted at 15° in its plane relative to the floor without adjusting the existing angulation of the head of the bed. | Pplat increased by a mean of 2.8±3.3 cmH_2_O (p = 0.01). Driving pressure increased by a mean of 2.9±3.3 cmH_2_O (p = 0.01). C_RS_ decreased by 3.4 ±3.7 mL/cmH_2_O (p = 0.01). | The C_RS_ decreased with bed inclination, increasing the driving pressure. |
| Marrazzo et al. Journal of clinical medicine 2023 (11) | C-ARDS patients  (n= 12) | Comparison of PEEP setting between supine-flat position at 0° and semi-recumbent position at 40° | Measured variables after 30 minutes at "Best" PEEP using electrical impedance tomography:  The PEEP was 14±2 cmH_2_O in the supine-flat position vs. 9±2 cmH_2_O in the semi-recumbent position (p < 0.001). | The trunk inclination angle significantly influences respiratory mechanics. Specifically, lower PEEP levels are required in a semi-reclined position than in a supine-flat position to optimize these mechanics. |
| Marrazzo et al. Respir Care. 2023 (1) | C-ARDS patients  (n= 15) | From supine-flat (0°) to semirecumbent (40°) position | C_RS_ decreased from 42 [35–51] to 31 [24–35] mL/cmH_2_O (p <0.001). Lung compliance decreased from 52 [41 – 80] to 45 [31–56] mL/cmH_2_O (p<0.01). The chest-wall compliance decreased from 266 [148–326] to 125 [95–146] mL/cmH_2_O (p<0.01). The driving pressure increased from 9±2 to 13±3 cmH_2_O (p<0.01). | Changes in body position from supine-flat to semi-recumbent impaired respiratory mechanics. |
| Benites et al. Intensive Care Med Experimental 2023 (3) | C-ARDS patients (n= 18),  ARDS (n=4) | From supine-flat at 10° to semirecumbent position at 45° | Tidal volume decreased from 433±84 to 371±76 mL (p < 0.001). C_RS_ decreased from 41±12 to 34±10 mL/cmH_2_O (p< 0.001). | Trunk inclination from 10° to 45° generated a decrease in tidal volume and C_RS_ |
| Pearce et al.  Crit Care Explor  2023 (15) | ARDS patients (n= 14) | From supine at 0° to semirecumbent head-up at 35-40° | C_RS_ decreased from 33±21 to 26±14 mL/cmH_2_O p= 0.005  Driving pressure increased from 14±6 to 17±7 cm H_2_O p<0.001 | Changes in body position from supine-flat to semi-recumbent impaired respiratory mechanics. |
| Bihari at al. Chest 2023  (14) | 40 patients with ARDS (20 obese, 20 non-obese) | From supine at 0° to semirecumbent head-up at 35-40° | Obese patients: Lung elastance increased from 20.5 ± 4.1 cmH_2_O/L to 24.1 ± 5.4 cmH_2_O/L (p <0.001). Chest wall elastance increased from 7.0 ± 1.9 cmH_2_O/L to 8.8 ± 2.3 cmH2O/L (p<0.001). Lung driving pressure increased from 8.3 ± 1.4 cmH_2_O to 10.5 ± 1.7 cmH_2_O (p<0.001). Non-obese patients: Lung elastance did not change significantly (p = 0.25) from 21.1 ± 5.1 cmH_2_O/L to 20.8 ± 4.9 cmH_2_O/L. Chest wall elastance increased from 7.6 ± 2.6 cmH_2_O/L to 7.9 ± 2.5 cmH_2_O/L (p<0.001). | In obese ARDS patients, the supine-flat position provided improved lung and chest wall elastance compared with the semi-recumbent position. In non-obese patients, only chest wall elastance was higher in the supine flat position than in the semi-recumbent position. |

## e-Table 4. Change in thoracic inclination and its effects on oxygenation

| Study | Patient Population  (n= 165) | Trunk Inclination | Outcome | conclusion |
| --- | --- | --- | --- | --- |
| Richard et al. Richard et al. Intensive Care Med 2006 (6) | ARDS patients (n=16) | Supine-flat (0°) to Semirecumbent head-up (45°) with lower limbs down at 45°. | PaO2 increased from 94 ± 33 mmHg to 142 ± 49 p < 0.0032). In 11 patients, PaO2 increased by more than 40%  In nonresponders (n 4), PaO2 did not significantly change (95 ± 2% vs. 97 ± 2%; p = 0.197) | The vertical position is a simple and well-tolerated method that can enhance oxygenation in selected ARDS patients. This positive impact is likely due to a variety of physiological factors, with lung recruitment playing a significant role in some patients. |
| Delllamonica et al. Intensive Care Med 2013 (2) | ARDS patients  (n=40) | From 15° to 45° of trunk inclination. | PaO2/FiO2 raised from 130 (110 - 151) to 210 (175 -222) mmHg only in responders (n=13).  In nonresponders (n=27), PaO2/FiO2 declined slightly from 132 (117 - 185) to 136 (111 - 178) mmHg and PaO2 from 91 (80 - 109) to 90 (83 - 107) mmHg.  No correlation was found between trunk inclination change and improved oxygenation r2 = 0.07,  p = ns. | In the majority of ARDS patients studied, there was no change in the PaO2/FiO2 ratio. However, those who did show an improvement in PaO2/FiO2 also experienced a rapid and significant increase in lung volume. Specifically, these responders exhibited a greater increase in EELV/PBW compared to non-responders.  In general, no correlation was observed between the changes in EELV and oxygenation. |
| Marrazzo et al. Am J Respir Crit Care Med 2022 (10) | C-ARDS patients  (n= 20) | From semirecumbent head-up 40° to supine-flat 0° position | PaO2/FiO2 (mmHg) did not show significant changes  (145 (115–189) vs. 140 (102–175)) p=0.74 | In patients with C-ARDS, no significant changes were observed in postural changes from the semi-recumbent to the supine-flat position. |
| Marrazzo et al. Journal of clinical medicine 2023 (11) | C-ARDS patients  (n= 12) | Comparison of PEEP setting between supine flat position at 0° and semi-recumbent position at 40° | Variables Measured after 30 min at “Best” PEEP using EIT  PaO2:FiO2 in supine flat-position was 141 ± 46 and in semirecumbent position was 196 ± 99 0.02 | Improved oxygenation was observed in the semi-recumbent position, as indicated by an elevated PaO2:FiO2 ratio, despite a reduction in PEEP after optimization. A diminished tendency toward alveolar derecruitment was also recorded over the 30-minute observation period.  This observation was corroborated by the significant decrease in End-Expiratory Lung Impedance (EELI), which was noted in the supine-flat position, particularly in the dorsal lung regions. In contrast, no change or even a slight increase in EELI was detected when the patients were placed in the semi-recumbent position. A possible role of end-expiratory transpulmonary pressure in these phenomena can be conjectured. |
| Marrazzo et al. Respir Care. 2023 (1) | C-ARDS patients  (n= 15) | From semirecumbent head-up 40° to supine-flat 0° position | PaO2/FiO2 164 ± 65 151 ± 49 p= 0.23 | In patients with C-ARDS, no significant changes were observed in postural changes from the semi-recumbent to the supine-flat position. |
| Benites et al. Intensive Care Med Experimental 2023 (3) | C-ARDS patients (n= 18)  ARDS (n=4) | From semirecumbent head-up 45° to supine-flat 10° position | PaO2/FiO2 (mmHg) did not show significant changes (189 ± 33 vs 196 ± 34) p = 0.735 | In patients with C-ARDS and ARDS, no significant changes were observed in postural changes from the semi-recumbent to the supine-flat position. |
| Bihari at al. Chest 2023  (14 ) | 40 patients with ARDS (20 obese, 20 non-obese) | Semi-recumbent (40° head up) vs supine-flat (0°) | Obese ARDS patients:  PaO2/FIO2:  Supine: 132  Semi-recumbent: 133  P-value: 0.65  Non-Obese ARDS patients:  PaO2/FIO2:  Supine: 143  Semi-recumbent: 143  P-value: 0.72 | There was no significant difference in PaO2/FIO2 between the supine and semi-recumbent positions in either the obese or non-obese ARDS patients. |

## eTable 5. Effects of trunk inclination on PaCO2 and Ventilatory inefficiency

| **Study** | **Patient Population**  **(n= 149)** | **Trunk Inclination** | **Outcome** | **Conclusions** |
| --- | --- | --- | --- | --- |
| Delllamonica et al. Intensive Care Med 2013 (2) | ARDS patients  (n=40) | From supine-flat (15°) to semirecumbent (45°) position | PaCO_2_ levels: 39.0 [35 - 47] vs. 40 [35; 48] mmHg. | There was no significant variability in PaCO_2_ levels with changes in trunk inclination. |
| Marrazzo et al. Am J Respir Crit Care Med 2022 (10) | C-ARDS patients  (n= 20) | From supine-flat (0°) to semirecumbent (40°) position | PaCO2 increased from 50 [46–54] mmHg to 52 [47–57] mmHg. p < 0.001.  The ventilatory ratio (VR) increased from 1.68 [1.43–1.96] to 1.81 [1.47–2.02], p= 0.001). | In volume-controlled ventilation patients, significant variations in PaCO_2_ and VR levels were observed when the thoracic inclination angle was changed. The increases in PaCO2 and VR can be attributed to the increased lung stress generated when the trunk is inclined at a 40° angle. |
| Marrazzo et al. Respir Care. 2023 (1) | C-ARDS patients  (n= 15) | From supine-flat (0°) to semirecumbent (40°) position | PaCO_2_ increased from 47 ± 6 to 52 ± 8 mmHg p<0.001  VR from rose 1.58 ± 0.27 to 1.75 ± 0.37. | Patients with C-ARDS experienced increases in PaCO_2_ and VR when the bed inclination angle was changed from supine-flat to semi-reclined. Those involved in these increases suggest alveolar overdistention in a semi-inclined position. |
| Hoste et al.  J Intensive Care Med 2005 (7) | ARDS patients  (n=18) | From semirecumbent position to upright position | With changes in trunk inclination, PaCO2 did not show significant variations in the first hour of the study (47 (10.7) vs. 48 (9.4) mm Hg). After 12 h in the upright position, PaCO2 decreased to 43 (8.3) mmHg. | There was no change in PaCO_2_ when shifting from the semi-recumbent to the upright position. |
| Pearce et al. Crit Care Explor  2023 (15) | ARDS patients  (n= 13) | From supine at 0° to semirecumbent head-up at 35-40° | PaCO_2_ values increased from 48.0 ± 11.4 mmHg to 54.8 ± 23.0 mmHg. | In this study, the effects on PaCO_2_ could not be adequately evaluated because of the small number of patients with arterial blood gas data. |
| Benites et al. Intensive Care Med Experimental 2023 (3) | C-ARDS patients (n= 18),  ARDS (n=4) | From supine-flat at 10° to semirecumbent position at 45° | PaCO_2_ increased from 36 (±4) to 43 (±5) mmHg p < 0.001.  Expired CO_2_ per minute (VCO_2_, min) decreased from 227 (±38) to 191 (±34) mL/min^-1^. p <0.001.  Bohr's dead space increased from 0.41 (±0.06) to 0.49 (±0.07) p <0.001.  SnIII increased from 0.58 [0.49 – 1.03] to 1.45 [0.75 – 2.07] L^-1^ p <0.001.  VR increased from 1.43 (±0.31) to 1.66 (±0.39) p = 0.107 | Minute ventilation decreased primarily because of the lower tidal volume when patients were positioned at a 40° angle. This led to impairments in both the V̇CO2 and Bohr's dead space, suggesting lung overdistension. This effect can be explained by a notable increase in SnIII when the patients were at 40°, indicating a less efficient CO_2_ exchange. |
| Bihari at al. Chest 2023  (14) | 40 patients with ARDS (20 obese, 20 non-obese) | From supine at 0° to semirecumbent head-up at 35-40° | Obese ARDS patients:  PaCO_2_: Supine-flat: 48 (±6) mmHg. Semi-recumbent: 50 (±6) mmHg. P-value: <0.001  non-obese ARDS patients:  Supine-flat: 50 (±8) mmHg.  Semi-recumbent: 50 (±8) mmHg. P-value: 0.72 | In obese patients with ARDS, a significant increase in PaCO_2_ was recorded with a change in bed inclination from a supine-flat to semi-recumbent position. Strikingly, no changes in PaCO_2_ were observed in non-obese patients. |
| Marrazzo et al. Journal of clinical medicine 2023 (11) | C-ARDS patients  (n= 12) | Comparison of PEEP setting between supine-flat position at 0° and semi-recumbent position at 40° | PaCO_2_ measured in the supine flat position was 45 (± 4), and in the semi-recumbent position was 47 ± (5) mmHg. p= 0.19. | The PEEP settings were optimized for each body posture. Similar levels of PaCO_2_ were observed at both positions. Unlike other studies, patients in the semi-recumbent position did not show increases in PaCO_2_ because of lower PEEP requirements. These results support the hypothesis that alveolar overdistension may be critical in impaired CO_2_ when PEEP is not optimized in a semi-recumbent position. |

## Forest Plot: PaCO2

**Figure. Forest plot. Mean difference in PaCO2 in classic ARDS patients with adjustments in trunk inclination from supine-flat to semi-recumbent position.**

**
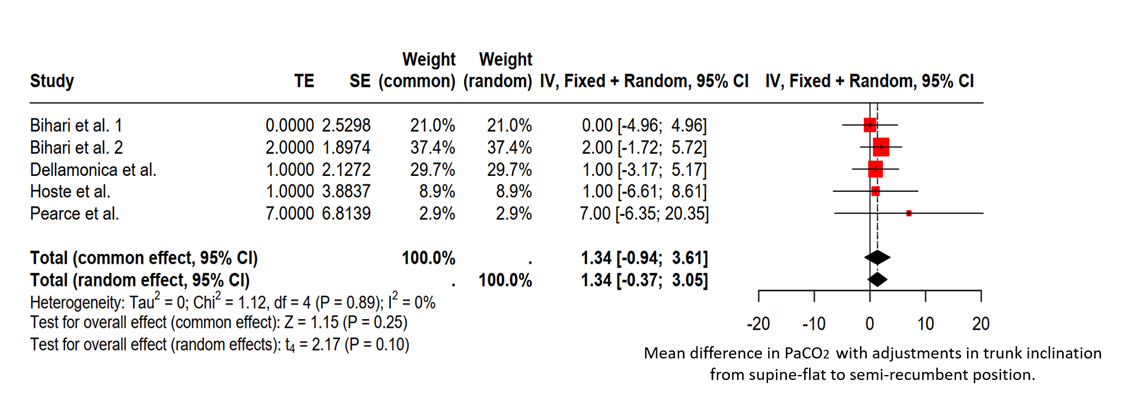
**

**Figure.** **Forest plot. Mean difference in PaCO2 in COVID-19-associated acute respiratory distress syndrome (C-ARDS) patients with adjustments in trunk inclination from supine-flat to semi-recumbent position.**

**
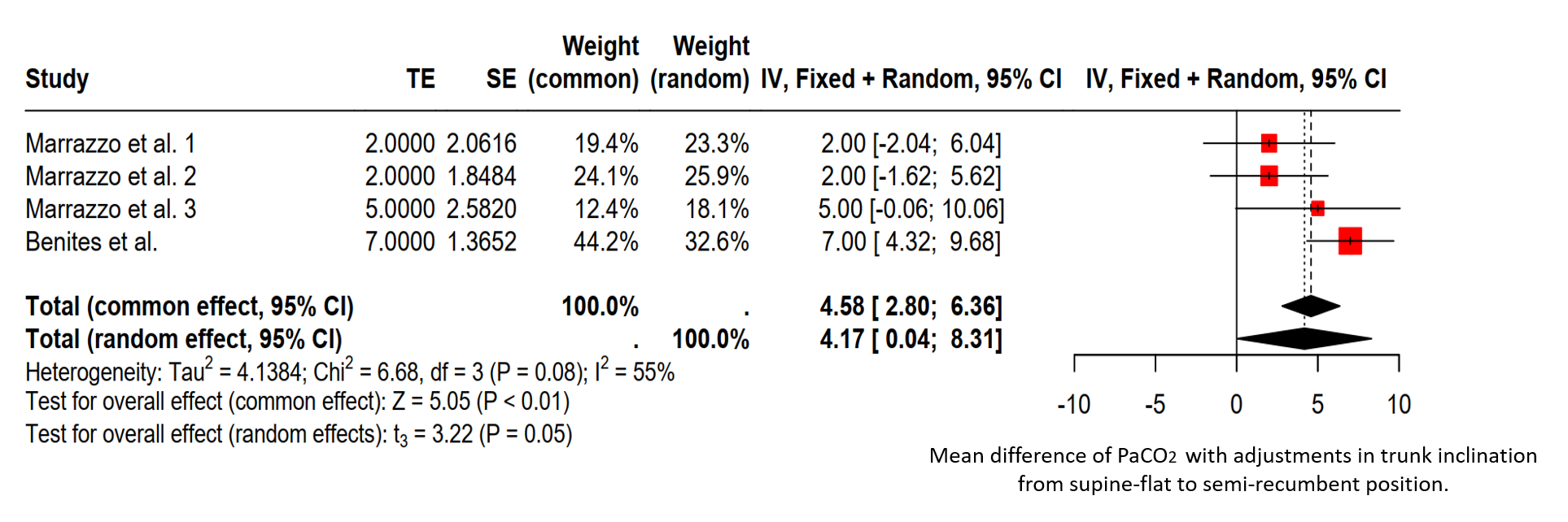
**

## Forest Plot: Compliance of respiratory system (C_RS_)

**Figure. Forest plot. Mean difference in C_RS_  in classic ARDS patients with adjustments in trunk inclination from supine-flat to semi-recumbent position.**

**
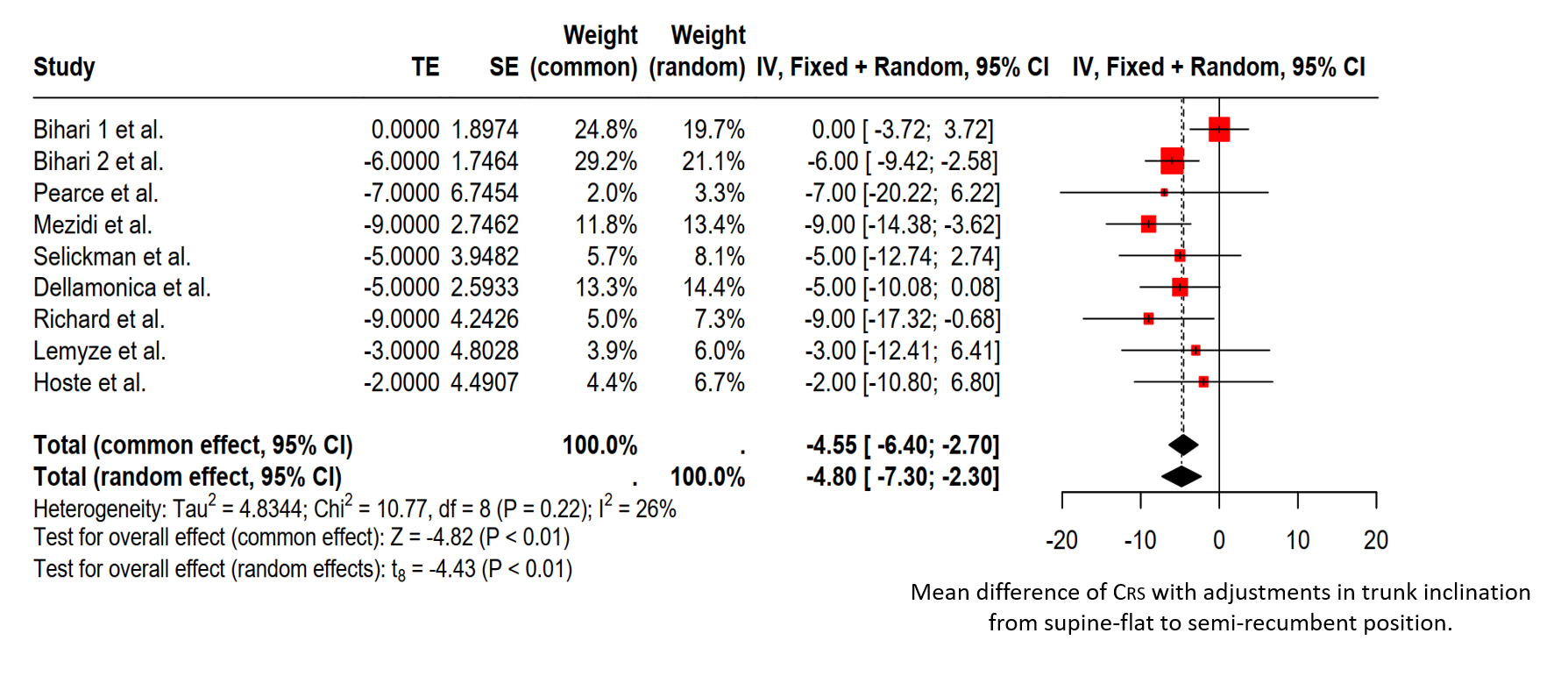
**

**Figure. Forest plot. Mean difference in C_RS_  in COVID-19-associated acute respiratory distress syndrome (C-ARDS) patients with adjustments in trunk inclination from supine-flat to semi-recumbent position.**

**
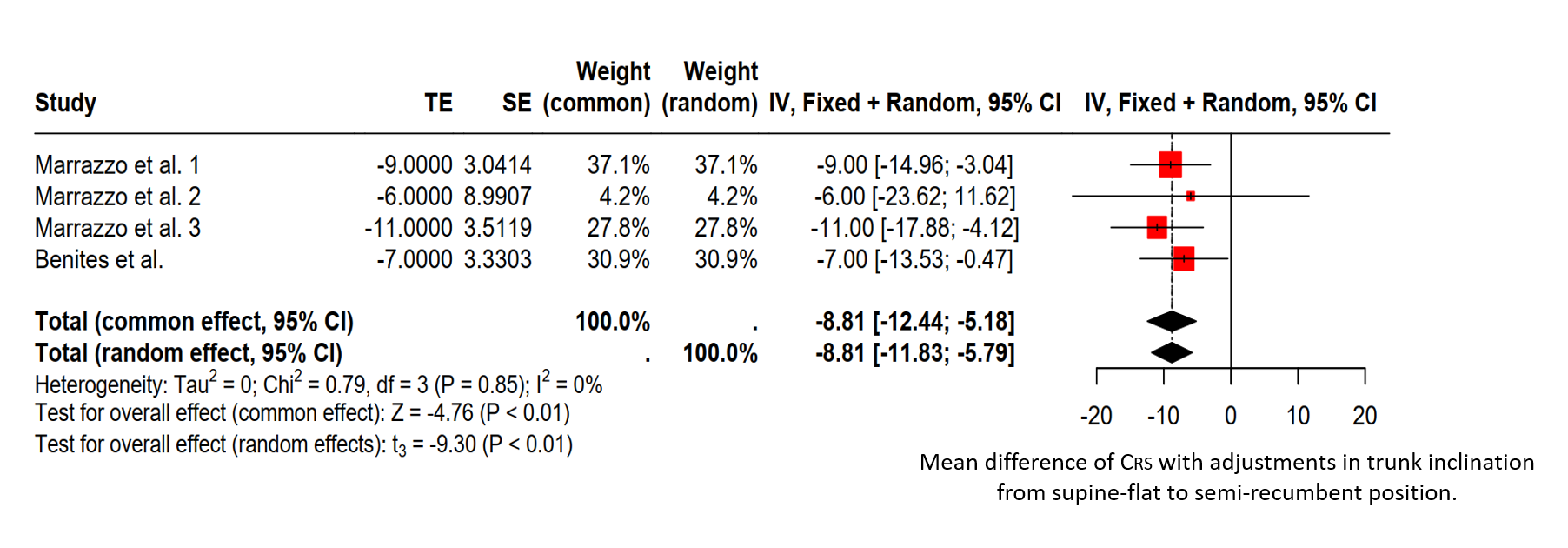
**

## Forest Plot: PaO2/FIO2

**Figure. Forest plot. Mean difference in PaO2/FIO2 in classic ARDS patients with adjustments in trunk inclination from supine-flat to semi-recumbent position.**

**
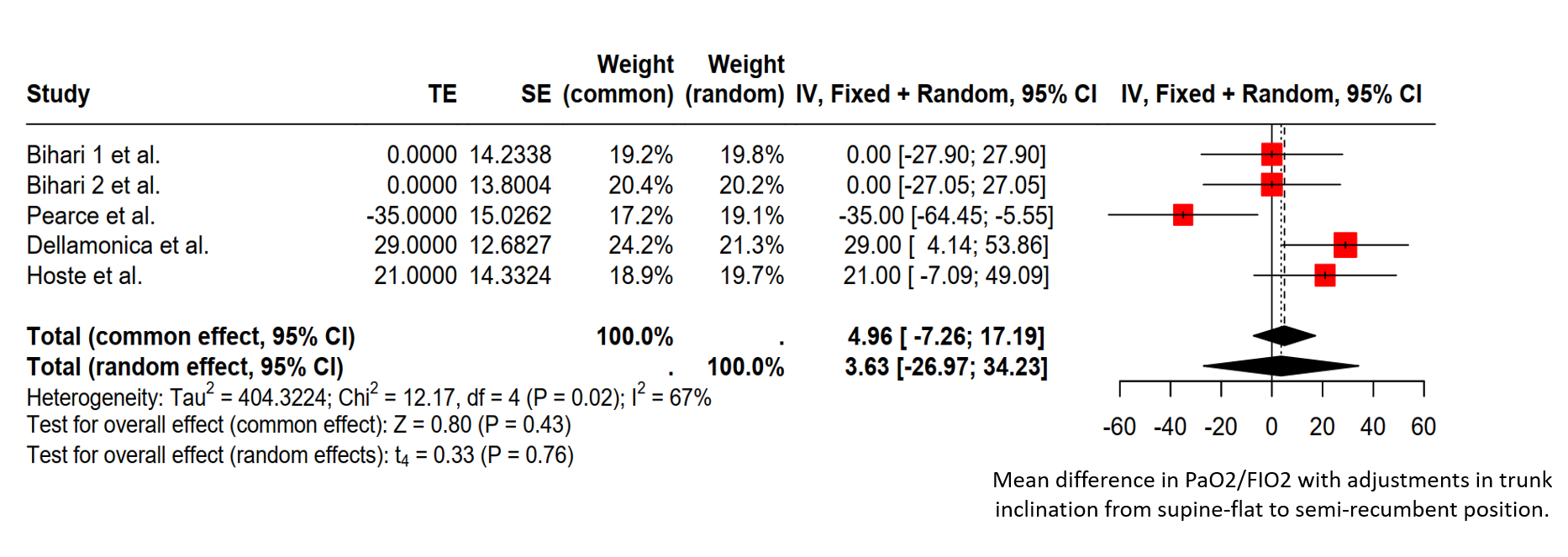
**

**Figure. Forest plot. Mean difference in PaO2/FIO2 in COVID-19-associated acute respiratory distress syndrome (C-ARDS) patients with adjustments in trunk inclination from supine-flat to semi-recumbent position.**

**
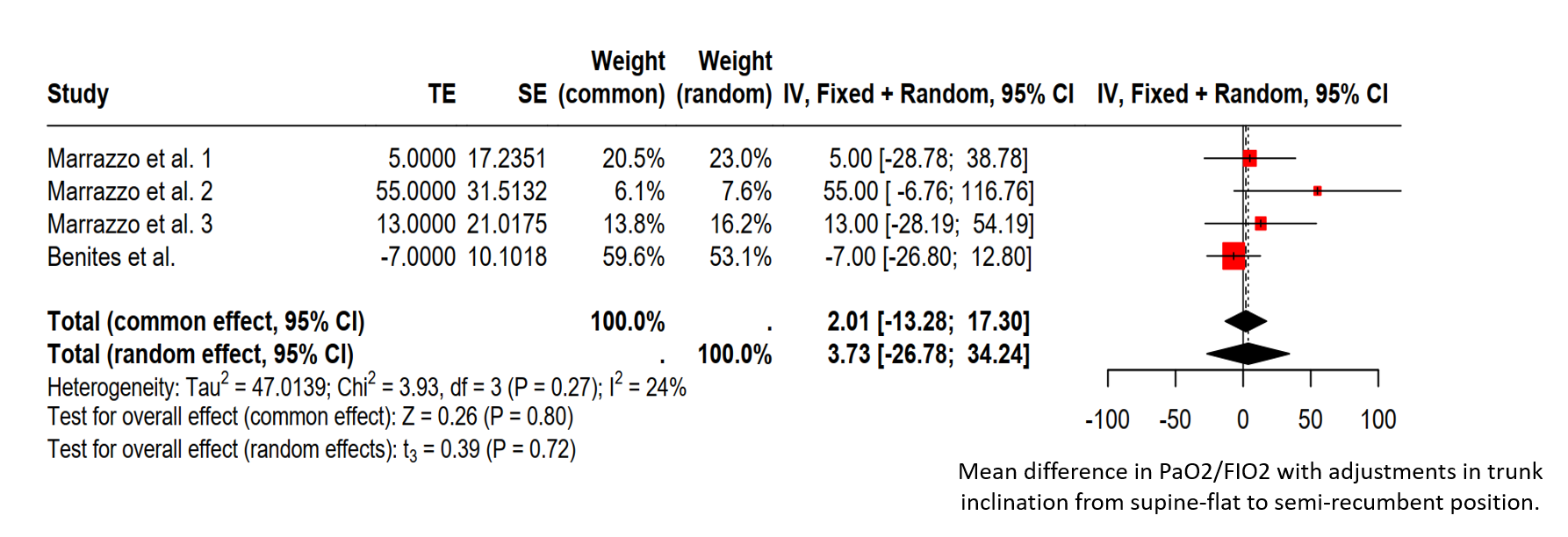
**

## Registered Search Strategy

**Pub med <2003 to 2023>**

(("Respiratory Insufficiency"[Mesh] OR "Respiratory Distress Syndrome"[Mesh] OR "respiratory failure" OR "acute respiratory distress syndrome" OR "ARDS" OR "COVID-19-associated acute respiratory distress syndrome" OR "COVID-19 ARDS") AND ("Sitting Position"[Mesh] OR "trunk inclination" OR "head up position" OR "reverse Trendelenburg position" OR "inclination angle" OR "vertical positioning" OR "seated position" OR "upright position" OR "inclination in supine position" OR "body position" OR "head-of-bed elevation") AND ("Respiratory Mechanics"[Mesh] OR "Pulmonary Gas Exchange"[Mesh] OR "ventilatory efficiency" OR "end-expiratory lung volume" OR "lung volume" OR "gas exchange" OR "oxygenation" OR “Ventilation distribution”))

**Search: Scopus on <2003 to 2023>**

TITLE-ABS-KEY ( ( "Respiratory Insufficiency" OR "Respiratory Distress Syndrome" OR "respiratory failure" OR "acute respiratory distress syndrome" OR "ARDS" OR "COVID-19-associated acute respiratory distress syndrome" OR "COVID-19 ARDS" ) AND ( "Sitting Position" OR "trunk inclination" OR "head up position" OR "reverse Trendelenburg position" OR "inclination angle" OR "vertical positioning" OR "seated position" OR "upright position" OR "inclination in supine position" OR "body position" OR "head-of-bed elevation" ) AND ( "Respiratory Mechanics" OR "Pulmonary Gas Exchange" OR "ventilatory efficiency" OR "end-expiratory lung volume" OR "lung volume" OR "gas exchange" OR "oxygenation" OR "Ventilation distribution" ) )

**Search: Cochrane on <2003 to 2023>**

48 Trials matching *( ( "Respiratory Insufficiency" OR "Respiratory Distress Syndrome" OR "respiratory failure" OR "acute respiratory distress syndrome" OR "ARDS" OR "COVID-19-associated acute respiratory distress syndrome" OR "COVID-19 ARDS" ) AND ( "Sitting Position" OR "trunk inclination" OR "head up position" OR "reverse Trendelenburg position" OR "inclination angle" OR "vertical positioning" OR "seated position" OR "upright position" OR "inclination in supine position" OR "body position" OR "head-of-bed elevation" ) AND ( "Respiratory Mechanics" OR "Pulmonary Gas Exchange" OR "ventilatory efficiency" OR "end-expiratory lung volume" OR "lung volume" OR "gas exchange" OR "oxygenation" OR "Ventilation distribution" ) )

Search Strategy.

Pubmed: n= 56

Cochrane: n= 48

Scopus: n= 162

Total = 266 studies identified

**Analysis of Duplicates by study year**

**Duplicate records removed 2003 – 2023 n = 46. “highlighted in grey.”**

- **2023 - Duplicate records removed: 5 (five)**

PubMed

1. Effects of changes in trunk inclination on ventilatory efficiency in ARDS patients: quasi-experimental study.
2. Facilitated Intubation: Time to Re-examine an Old Technique With Its Associated Risks Mitigated by New Technology
3. Ventilation Distribution During Changes in Trunk Inclination in Patients With ARDS
4. The impact of semi-upright position on severity of sleep disordered breathing in patients with obstructive sleep apnea: a two-arm, prospective, randomized controlled trial
5. Curiosity, Opportunity, and Luck: Were the 1970s Different?
6. PEEP Titration Is Markedly Affected by Trunk Inclination in Mechanically Ventilated Patients with COVID-19 ARDS: A Physiologic, Cross-Over Study
7. Personalized ventilatory strategy based on lung recruitablity in COVID-19-associated acute respiratory distress syndrome: a prospective clinical study
8. Real-time effects of lateral positioning on regional ventilation and perfusion in an experimental model of acute respiratory distress syndrome.
9. Changes of blood gas analysis in moderate-to-severe acute respiratory distress syndrome patients during long-term prone position ventilation: a retrospective cohort study
10. Pressure ulcers after prone positioning in patients undergoing extracorporeal membrane oxygenation: A cross-sectional study
11. Impact of Supine Versus Semirecumbent Body Posture on the Distribution of Ventilation in Acute Respiratory Distress Syndrome. Crit Care Explor. 2023 Dec 1;5(12):e1014.

Cochrane

1. Examination of respiratory mechanics with trunk inclination in obese and non-obese ARDS patients
2. Effects of bending and prone position ventilation on respiratory and circulatory functions in patients with ARDS

Scopus

1. Effects of changes in trunk inclination on ventilatory efficiency in ARDS patients: quasi-experimental study
2. Personalized ventilatory strategy based on lung recruitablity in COVID-19-associated acute respiratory distress syndrome: a prospective clinical study
3. Curiosity, Opportunity, and Luck: Were the 1970s Different?
4. Robust Non-Contact Monitoring of Respiratory Rate using a Depth Camera
5. PEEP Titration Is Markedly Affected by Trunk Inclination in Mechanically Ventilated Patients with COVID-19 ARDS: A Physiologic, Cross-Over Study
6. Preoperative cerebral oxygenation in high-risk noncardiac surgical patients: an observational study on postoperative mortality and complications
7. Regional ventilation in spontaneously breathing COVID-19 patients during postural maneuvers assessed by electrical impedance tomography
8. Lung ultrasound to predict gas-exchange response to prone positioning in COVID-19 patients: A prospective study in pilot and confirmation cohorts
9. Real-time effects of lateral positioning on regional ventilation and perfusion in an experimental model of acute respiratory distress syndrome
10. Pressure ulcers after prone positioning in patients undergoing extracorporeal membrane oxygenation: A cross-sectional study
11. Physio-Metabolic Monitoring via Breath Employing Real-Time Mass Spectrometry: Importance, Challenges, Potentials, and Pitfalls

- **2022 - Duplicate records removed: 9 (nine)**

Pub Med

1. Positive end-expiratory pressure and prone position alter the capacity of force generation from diaphragm in acute respiratory distress syndrome: an animal experiment
2. Paradoxical Positioning: Does "Head Up" Always Improve Mechanics and Lung Protection?
3. Effect of body position on the redistribution of regional lung aeration during invasive and non-invasive ventilation of COVID-19 patients.
4. Finite Element Modeling of Pulmonary Mechanics in Severe Acute Respiratory Distress Syndrome: Explaining the Inclination Angle?
5. In Vitro and In Vivo Feasibility Study for a Portable VV-ECMO and ECCO2R System.
6. Sequential lateral positioning as a new lung recruitment maneuver: an exploratory study in early mechanically ventilated Covid-19 ARDS patients.
7. Effects of Trunk Inclination on Respiratory Mechanics in Patients with COVID-19-associated Acute Respiratory Distress Syndrome: Let's Always Report the Angle!
8. Prolonged Continuous Monitoring of Regional Lung Function in Infants with Respiratory Failure.

Cochrane

1. Smartphone-guided Self-prone Positioning versus Usual Care in Non-Intubated Hospital Ward Patients with COVID-19: a Pragmatic Randomized Clinical Trial
2. The protective versus conventional ventilation during laparoscopic surgery trial
3. HFNC Versus Oxygen Face Mask on Postoperative Pulmonery Complications
4. Effect of sufentanil in saddle block on delivery parameters
5. Smartphone-Guided Self-prone Positioning vs Usual Care in Nonintubated Hospital Ward Patients With COVID-19: a Pragmatic Randomized Clinical Trial
6. Effect of a FLUid Bolus or a Low Dose VAsopressor Infusion on Cardiovascular Collapse Among Critically Ill Adults Undergoing Tracheal Intubation
7. Smartphone-Guided Self-prone Positioning vs Usual Care in Nonintubated Hospital Ward Patients With COVID-19: a Pragmatic Randomized Clinical Trial
8. Analysis of the Effects of Humidified High Flow Nasal Oxygen Therapy Combined with Noninvasive Mechanical Ventilation on Treatment OutcomeS

Scopus

1. Positive end-expiratory pressure and prone position alter the capacity of force generation from diaphragm in acute respiratory distress syndrome: an animal experiment
2. Chest wall loading in the ICU: pushes, weights, and positions
3. Capnodynamic monitoring of lung volume and blood flow in response to increased positive end-expiratory pressure in moderate to severe COVID-19 pneumonia: an observational study
4. Effect of body position on the redistribution of regional lung aeration during invasive and non-invasive ventilation of COVID-19 patients
5. Sequential lateral positioning as a new lung recruitment maneuver: an exploratory study in early mechanically ventilated Covid-19 ARDS patients
6. Effects of prone and lateral positioning alternate in high-flow nasal cannula patients with severe COVID-19
7. Paradoxical Positioning: Does Head Up Always Improve Mechanics and Lung Protection?
8. Prone Positioning for Patients with COVID-19-Associated Acute Respiratory Distress Syndrome
9. Finite Element Modeling of Pulmonary Mechanics in Severe Acute Respiratory Distress Syndrome: Explaining the Inclination Angle?
10. COVID-19-Related ARDS: Key Mechanistic Features and Treatments
11. Therapeutic benefits of proning to improve pulmonary gas exchange in severe respiratory failure: focus on fundamentals of physiology
12. Prone positioning redistributes gravitational stress in the lung in normal conditions and in simulations of oedema
13. Positioning for acute respiratory distress in hospitalised infants and children
14. Prolonged Continuous Monitoring of Regional Lung Function in Infants with Respiratory Failure
15. Effects of Trunk Inclination on Respiratory Mechanics in Patients with COVID-19–associated Acute Respiratory Distress Syndrome: Let’s Always Report the Angle!
16. Match Me If You Can: The Relationship between Ventilation and Perfusion with Position Changes in Nonhomogenous Lung Injury
17. In Vitro and In Vivo Feasibility Study for a Portable VV-ECMO and ECCO2 R System
18. Advanced respiratory monitoring in mechanically ventilated patients with coronavirus disease 2019- associated acute respiratory distress syndrome
19. Ventilatory Management of the Patient with Severe Obesity
20. Independent lung ventilation for the management of acute allograft rejection after single-lung transplantation for end-stage emphysema
21. Prolonged non-invasive respiratory supports in a patient with COVID-19 and severe acute hypoxemic respiratory failure: A case report
22. The Effects of Rso2 and PI Monitoring Images on the Treatment of Premature Infants Based on Deep Learning
23. Combining Non-invasive Ventilation with timed position change in the Emergency Department to improve oxygenation and outcomes in patients with COVID-19: A prospective analysis from a low resource setup
24. Perioperative Pulmonary Atelectasis: Part II. Clinical Implications

- **2021 - Duplicate records removed: 2 (two)**

PubMEd

1. Effect of Position Change From the Bed to a Wheelchair on the Regional Ventilation Distribution Assessed by Electrical Impedance Tomography in Patients With Respiratory Failure.
2. Diminishing Efficacy of Prone Positioning With Late Application in Evolving Lung Injury.

Cochrane

1. Awake Prone Positioning in COVID-19 Suspects With Hypoxemic Respiratory Failure
2. The effects of different body positions during ventilation on the cardiopulmonary function, blood gas, and inflammation indicators in severe acute respiratory distress syndrome patients (lateral position)

Scopus

1. Perioperative management of emergent cesarean section in a patient with peripartum cardiomyopathy and orthopnea: a case report
2. Continuous Lower Abdominal Compression as a Therapeutic Intervention in COVID-19 ARDS
3. Platypnea–orthodeoxia syndrome associated with COVID-19 pneumonia: a case report
4. SARS-CoV-2 pneumonia succesfully treated with cpap and cycles of tripod position: a case report
5. Effect of Position Change From the Bed to a Wheelchair on the Regional Ventilation Distribution Assessed by Electrical Impedance Tomography in Patients With Respiratory Failure
6. Target and goal mismatch during mechanical ventilation in COVID-19 patients
7. Paradoxical Effect of Chest Wall Compression on Respiratory System Compliance: A Multicenter Case Series of Patients With ARDS, With Multimodal Assessment
8. Diminishing Efficacy of Prone Positioning With Late Application in Evolving Lung Injury
9. A case of spontaneous rectus sheath hematoma induced by lateral semi-prone positional changes during extracorporeal membrane oxygenation
10. Effects of prone and lateral position in non-intubated patients with 2019 Novel Coronavirus (COVID-19) pneumonia
11. The Obese Patient With Acute Respiratory Failure
12. Impact of Prone Position on 12-Lead Electrocardiogram in Healthy Adults: A Comparison Study with Standard Electrocardiogram

- **2020 - Duplicate records removed: 5 (Five)**

Pub Med

1. How to ventilate obese patients in the ICU
2. A primer on proning in the emergency department
3. Management and experience of postural placement in postoperative mechanical ventilation of newborns.
4. [A strange case of acute respiratory failure: the platypnea-orthodeoxia syndrome].
5. Sources of variability in expiratory flow profiles during sleep in healthy young children
6. Determinants of the esophageal-pleural pressure relationship in humans.

Cochrane

1. Sargramostim in patients with acute hypoxic respiratory failure due to COVID-19
2. Incline Positioning in COVID-19 Patients for Improvement in Oxygen Saturation
3. Management and experience of postural placement in postoperative mechanical ventilation of newborns

Scopus

1. A primer on proning in the emergency department
2. How to ventilate obese patients in the ICU
3. Early application of prone position for management of Covid-19 patients
4. Management and experience of postural placement in postoperative mechanical ventilation of newborns
5. A strange case of acute respiratory failure: The platypnea-orthodeoxia syndrome | Un insolito caso di insufficienza respiratoria acuta: La sindrome platipnea-ortodeossia
6. COVID-19 associated pulmonary aspergillosis
7. Treating hypoxemic patients with SARS-COV-2 pneumonia: Back to applied physiology
8. Finding best peep: A little at a time
9. Study on the extracorporeal membrane oxygenation inter-hospital transport during coronavirus disease 2019 epidemic: Based on the transport experience of 6 cases of severe H1N1 influenza virus pneumonia on extracorporeal membrane oxygenation
10. Oxygen therapy delivery and body position effects measured with electrical impedance tomography. (spontaneous breathing)
11. The effects of forward head posture on expiratory muscle strength in chronic neck pain patients: A cross-sectional study
12. Determinants of the esophageal-pleural pressure relationship in humans

- **2019 - Duplicate records removed: 1 (one)**

Pub Med

1. Effect of body position and inclination in supine and prone position on respiratory mechanics in acute respiratory distress syndrome.

Cochrane

1. Early Use of Prone Position in ECMO for Severe ARDS
2. Pharmacodynamic interaction of remifentanil and dexmedetomidine on depth of sedation and tolerance of laryngoscopy

Scopus

1. Extreme obesity—particular aspect of invasive and noninvasive ventilation | Adipositas (permagna) – Besonderheiten bei der invasiven und nichtinvasiven Beatmung
2. Airway Closure during Surgical Pneumoperitoneum in Obese Patients
3. Sepsis-induced heparin resistance during extracorporeal membrane oxygenation
4. ARDS in Obese Patients: Specificities and Management
5. Effect of body position and inclination in supine and prone position on respiratory mechanics in acute respiratory distress syndrome

- **2018 - Duplicate records removed: 4 (four)**

Pub Med

1. Building on the Shoulders of Giants: Is the use of Early Spontaneous Ventilation in the Setting of Severe Diffuse Acute Respiratory Distress Syndrome Actually Heretical?
2. Effects of High-Flow Nasal Cannula on End-Expiratory Lung Impedance in Semi-Seated Healthy Subjects.
3. The obesity supine death syndrome (OSDS).
4. Intraoperative Ventilation of Morbidly Obese Patients Guided by Transpulmonary Pressure

Cochrane

1. Intraoperative Ventilation of Morbidly Obese Patients Guided by Transpulmonary Pressure
2. Oral levosimendan in ALS: the REFALS phase 3 study design
3. Effect of Inspiratory Muscle Training and Early Mobilization Program on Weaning of Mechanical Ventilation in Critically Ill Patients
4. CO2 Clearance During Noninvasive Ventilation (NIV)

Scopus

1. Expiratory Flow Limitation During Mechanical Ventilation
2. Effects of high-flow nasal cannula on end-expiratory lung impedance in semi-seated healthy subjects
3. The obesity supine death syndrome (OSDS)
4. Building on the shoulders of giants: Is the use of early spontaneous ventilation in the setting of severe diffuse acute respiratory distress syndrome actually heretical?
5. Outcomes of extracorporeal membrane oxygenation in adult patients with hypoxemic respiratory failure refractory to mechanical ventilation
6. Intraoperative Ventilation of Morbidly Obese Patients Guided by Transpulmonary Pressure **
7. Electrical impedance tomography as possible guidance for individual positioning of patients with multiple lung injury (spontaneous breathing)

- **2017 - Duplicate records removed: 0 (zero)**

Pub Med

Cochrane

1. A Quasi-Experimental, Before-After Trial Examining the Impact of an Emergency Department Mechanical Ventilator Protocol on Clinical Outcomes and Lung-Protective Ventilation in Acute Respiratory Distress Syndrome
2. Lung-Protective Ventilation Initiated in the Emergency Department (LOV-ED): a Quasi-Experimental, Before-After Trial
3. The evaluation of the astral ivaps autoepap treatment algorithm

Scopus

1. Extracorporeal membrane oxygenation in spina bifida and (H1N1)-induced acute respiratory distress syndrome
2. Should we use driving pressure to set tidal volume?

- **2016 - Duplicate records removed: 2 (two)**

Pub Med

1. [What Should We Know about Respiratory Physiology for the Optimal Anesthesia Management?]
2. A phase IV, single-center, crossover evaluation of the efficacy of an external nasal dilator strip in children with nasal congestion.
3. Lateral positioning for critically ill adult patients.
4. Peak nasal inspiratory flow and peak expiratory flow. Upright and sitting values in an adult population

Cochrane

1. The effect of sugammadex on respiratory complications and patient satisfaction following surgery
2. HFCWO on Pneumonic Respiratory Failure

Scopus

1. Effect of exercise on vital capacity in different posture of young Indian subjects
2. Biotrauma and Ventilator-Induced Lung Injury: Clinical Implications
3. Mechanisms of orthopnea in stable obese subjects
4. Lateral positioning for critically ill adult patients
5. What should we know about respiratory physiology for the optimal anesthesia management?
6. Should we embrace the open lung approach?

- **2015 - Duplicate records removed: 1 (one)**

Pub Med

1. Preoxygenation and general anesthesia: a review.

Cochrane

1. Short Term Physiological Effects of Nasal High Flow Oxygen on Respiratory Mechanics
2. Lung Volume Recruitment in Neuromuscular Disease: can ‘breath-stacking’ improve lung function, respiratory symptoms and quality of life for people with neuromuscular disease?

Scopus

1. S2e guideline: positioning and early mobilisation in prophylaxis or therapy of pulmonary disorders: Revision 2015: S2e guideline of the German Society of Anaesthesiology and Intensive Care Medicine (DGAI) | S2e-Leitlinie
2. Advantages of the prone position for minimally invasive esophagectomy in comparison to the left decubitus position: better oxygenation after minimally invasive esophagectomy
3. A comprehensive review of prone position in ARDS
4. Awake’ extracorporeal membrane oxygenation requires adequate lower body muscle training and mobilisation as successful bridge to lung transplant
5. Preoxygenation and general anesthesia: A review

- **2014 - Duplicate records removed: 1 (one)**

Pub Med

1. A comparison of supine and prone positioning on improves arterial oxygenation in premature neonates.
2. Head-of-bed elevation improves end-expiratory lung volumes in mechanically ventilated subjects: a prospective observational study. Respir Care

Cochrane

1. Initiation of home mechanical ventilation at home in a selectve group of patients with chronic hypercapnic respiratory failure in the Netherlands
2. 28th Annual Meeting of the European Association of Cardiothoracic Anaesthesiologists, EACTA 2013
3. Ischemic preconditioning eliminates positional changes in oxygen saturation during hypoxia

Scopus

1. Should we prone cardiac surgery patients with acute respiratory distress syndrome?
2. Body position and oxygenation: An intriguing relationship
3. A comparison of supine and prone positioning on improves arterial oxygenation in premature neonates
4. Sleep and breathing
5. Positioning in mechanical ventilation | Lagerungstherapie bei beatmeten Intensivpatienten

- **2013 - Duplicate records removed: 4 (four)**

Pub Med

1. Ventilatory strategies and supportive care in acute respiratory distress syndrome.
2. Body position changes redistribute lung computed-tomographic density in patients with acute respiratory failure: impact and clinical fallout through the following 20 years.
3. Effect of different seated positions on lung volume and oxygenation in acute respiratory distress syndrome.
4. Effect of body position on ventilation distribution in ventilated preterm infants.

Cochrane

1. Effect of open and closed suction on Ventilator-associated Pneumonia and hemodynamic status
2. Effect of body position on ventilation distribution in ventilated preterm infants

Scopus

1. Is All on the Level? Hemodynamics during Supine versus Prone Ventilation
2. Effets de la ventilation en position verticale. À propos d'un patient en réanimation | Ventilation effects in vertical position. Case report on patient in intensive care unit
3. Ventilatory strategies and supportive care in acute respiratory distress syndrome
4. Effects of sitting position and applied positive end-expiratory pressure on respiratory mechanics of critically ill obese patients receiving mechanical ventilation
5. Body position changes redistribute lung computed-tomographic density in patients with acute respiratory failure: Impact and clinical fallout through the following 20 years
6. Erratum: Effect of different seated positions on lung volume and oxygenation in acute respiratory distress syndrome
7. Effect of different seated positions on lung volume and oxygenation in acute respiratory distress syndrome
8. Effect of body position on ventilation distribution in ventilated preterm infants
9. Surviving sepsis campaign: International guidelines for management of severe sepsis and septic shock, 2012
10. High-frequency oscillatory ventilation
11. Acute respiratory distress syndrome

- **2012 - Duplicate records removed: 1 (one)**

Pub Med

1. Platypnea-orthodeoxia syndrome related to right hemidiaphragmatic elevation and a 'stretched' patent foramen ovale.

Cochrane

Scopus

1. Continuous lateral rotational therapy and systemic inflammatory response in posttraumatic acute lung injury: Results from a prospective randomised study
2. Perioperative management of the severely obese patient: A selective pathophysiological review
3. Relationship between regional lung compliance and ventilation homogeneity in the supine and prone position
4. Management of the critically ill obstetric patient
5. Left tilt position for easy extracorporeal membrane oxygenation cannula insertion in late pregnancy patients
6. Left tilt position for cardiopulmonary bypass in parturient patients
7. Prone positioning for patients with ARDS: Although not common in ICUs, this therapy may help improve oxygenation. Here's what you need to know
8. Platypnea-orthodeoxia syndrome related to right hemidiaphragmatic elevation and a 'stretched' patent foramen ovale
9. Positioning for acute respiratory distress in hospitalised infants and children.
10. Evident-based nursing of a ventilation patient with acute respiratory distress syndrome: Exploring a reasonable position
11. Early detection of deteriorating ventilation: Prevention is better than cure!

- **2011 - Duplicate records removed: 1 (one)**

Pub Med

1. Short-term effects of combining upright and prone positions in patients with ARDS: a prospective randomized study.

Cochrane

1. Short-term effects of combining upright and prone positions in patients with ARDS: a prospective randomized study

Scopus

1. Positioning of patients with acute respiratory distress syndrome: Combining prone and upright makes sense
2. Acute respiratory distress syndrome and acute lung injury
3. Physiology of gas exchange during anaesthesia
4. Does positive end-expiratory pressure improve CO2 exchange in controlled ventilation of acute airflow obstruction?
5. Short-term effects of combining upright and prone positions in patients with ARDS: a prospective randomized study.

- **2010 - Duplicate records removed: 1 (one)**

Pub Med

Cochrane

1. The effect of different positioning after lung recruitment with CPAP hold maneuver on oxygenation and lung mechanics in acute respiratory distress syndrome
2. Prone positioning improves survival in severe ARDS: a pathophysiologic review and individual patient meta-analysis

Scopus

1. The effect of different positioning after lung recruitment with CPAP hold maneuver on oxygenation and lung mechanics in acute respiratory distress syndrome
2. Severe hypoxemic respiratory failure: Part 2-nonventilatory strategies
3. Abdominal volume contribution to tidal volume as an early indicator of respiratory impairment in Duchenne muscular dystrophy
4. Intra-abdominal hypertension, prone ventilation, and abdominal suspension
5. Prone positioning in patients with acute respiratory distress syndrome - In reply [2]
6. Prevalence and prognosis of shunting across patent foramen ovale during acute respiratory distress syndrome
7. Pharmacotherapy for prevention and treatment of acute respiratory distress syndrome: Current and experimental approaches

- **2009 - Duplicate records removed: 1 (one)**

Pub Med

1. Upright position mechanical ventilation: an alternative strategy for ALI/ARDS patients?

Cochrane

1. Effect of early prone postioning on mortality in patients with severe and persistent Acute Respiratory Distress Syndrome. Proseva Study. Description of the research protocol

Scopus

1. Ventilating ARDS beyond PEEP and Tidal Volume
2. Upright position mechanical ventilation: An alternative strategy for ALI/ARDS patients?
3. Effect of intrapulmonary percussive ventilation on expiratory flow limitation in chronic obstructive pulmonary disease patients
4. Functional residual capacity measurements in ventilated patients: Measures, determinants, clinical implications and perspectives
5. Extended prone position ventilation in severe acute respiratory distress syndrome: A pilot feasibility study
6. Prone positioning in hypoxemic respiratory failure: Meta-analysis of randomized controlled trials
7. Successful treatment of severe hepatopulmonary syndrome with a sequential use of TIPS placement and liver transplantation
8. Surgical techniques: Lung transplant and lung volume reduction

- **2008 - Duplicate records removed: 0 (zero)**

Pub Med

Cochrane

Scopus

1. Nasal reflexes: Implications for exercise, breathing, and sex
2. Effect of recruitment and body positioning on lung volume and oxygenation in acute lung injury model
3. BTS guideline for emergency oxygen use in adult patients
4. Effects of prone position and positive end-expiratory pressure on lung perfusion and ventilation

- **2007 - Duplicate records removed: 0 (zero)**

Pub Med

Cochrane

1. Inhaled nitric oxide and prone position: how far they can improve oxygenation in pediatric patients with acute respiratory distress syndrome?
2. Prone position in early and severe acute respiratory distress syndrome: a design for a definitive randomized controlled trial

Scopus

1. Association of body position with sleep architecture and respiratory disturbances in children with obstructive sleep apnea
2. Pneumomediastinum complicated by subclavian central venous catheterization in a severe thoracic trauma patient
3. Prolonged lateral steep position impairs respiratory mechanics during continuous lateral rotation therapy in respiratory failure
4. Cardiopulmonary interaction in heart failure

- **2006 - Duplicate records removed: 3 (three)**

Pub Med

1. The effect of lateral position on oxygenation in ARDS patients: a pilot study
2. Effects of vertical positioning on gas exchange and lung volumes in acute respiratory distress syndrome.
3. Cardiopulmonary interaction in heart failure

Cochrane

1. Ventilation in the prone position in patients with acute lung injury/acute respiratory distress syndrome
2. Clinical trial design--effect of prone positioning on clinical outcomes in infants and children with acute respiratory distress syndrome
3. Mechanical ventilation in the management of acute respiratory distress syndrome

Scopus

1. Acute Lung Injury and the Acute Respiratory Distress Syndrome: Challenges in Clinical Trial Design
2. The effect of lateral position on oxygenation in ARDS patients: A pilot study
3. Effects of vertical positioning on gas exchange and lung volumes in acute respiratory distress syndrome
4. Clinical trial design-effect of prone positioning on clinical outcomes in infants and children with acute respiratory distress syndrome
5. Positional therapy and polytrauma: Useful or dangerous? | Lagerungstherapie beim polytrauma: Sinnvoll oder gefährlich?

- **2005 - Duplicate records removed: 2 (two)**

Pub Med

1. Lateral decubitus position generates discomfort and worsens lung function in chronic heart failure.
2. [Platypnea-orthodeoxia syndrome, atrial septal aneurysm and right hemidiaphragmatic elevation with a right-to-left shunt through a patent foramen ovale].
3. Effect of body position on gas exchange in patients with idiopathic pulmonary alveolar proteinosis: no benefit of prone positioning.
4. Acute effects of upright position on gas exchange in patients with acute respiratory distress syndrome.

Cochrane

1. Effect of prone position on lung surfactant composition and function in multiple trauma patients with respiratory dysfunction

Scopus

1. Protective lung strategies during artificial ventilation in children
2. Large venous air embolism in the sitting position despite monitoring with transoesophageal echocardiography
3. Lung recruitment during mechanical positive pressure ventilation in the PICU: What can be learned from the literature?
4. Prone positioning in children with ARDS: Positive reflections on a negative clinical trial
5. Prone positioning: Do not turn it off!
6. Positioning for acute respiratory distress in hospitalised infants and children.
7. Acute effects of upright position on gas exchange in patients with acute respiratory distress syndrome
8. Effect of body position on gas exchange in patients with idiopathic pulmonary alveolar proteinosis: No benefit of prone positioning

- **2004 - Duplicate records removed: 1 (one)**

Pub Med

1. The role of body position and gravity in the symptoms and treatment of various medical diseases.

Cochrane

Scopus

1. The effect of various body positions on dynamic intrinsic PEEP during pressure support ventilation | Wielkość dynamicznego PEEP w czasie zmiany pozycji ciała u chorych w trakcie wentylacji wspomaganej
2. Comparison of incomplete (135°) and complete prone position (180°) in patients with acute respiratory distress syndrome. Results of a prospective, randomised trial | Vergleich von inkompletter (135°) und kompletter bauchlage (180°) beim schweren akuten lungenversagen. Ergebnisse einer prospektiven, randomisierten untersuchung
3. The role of body position and gravity in the symptoms and treatment of various medical diseases
4. Acute respiratory distress syndrome

- **2003 - Duplicate records removed: 2 (two)**

Pub Med

1. Comparison of positive end-expiratory pressure with reverse Trendelenburg position in morbidly obese patients undergoing bariatric surgery: effects on hemodynamics and pulmonary gas exchange.
2. Effect of alveolar recruitment maneuver in early acute respiratory distress syndrome according to antiderecruitment strategy, etiological category of diffuse lung injury, and body position of the patient.
3. The upper airway in pregnancy and pre-eclampsia.

Cochrane

1. ARDS management: high-frequency oscillatory ventilation as a new option
2. Evidence-based medicine in ARDS: what is really proven?
3. Decrease in Paco2 with prone position is predictive of improved outcome in acute respiratory distress syndrome
4. Effect of alveolar recruitment maneuver in early acute respiratory distress syndrome according to antiderecruitment strategy, etiological category of diffuse lung injury, and body position of the patient
5. Use of orthostasis during artificial pulmonary ventilation in patients with acute parenchymal pulmonary lesion

Scopus

1. Comparison of positive end-expiratory pressure with reverse Trendelenburg position in morbidly obese patients undergoing bariatric surgery: Effects on hemodynamics and pulmonary gas Exchange
2. Prone positioning of pediatric patients with ARDS results in improvement in oxygenation if maintained > 12 h daily
3. Combined effects of prone positioning and airway pressure release ventilation on gas exchange in patients with acute lung injury
4. Acute respiratory distress syndrome: Lessons from computed tomography of the whole lung
5. Efficacy of lung recruiting maneuvers: It's all relative
6. Effect of alveolar recruitment maneuver in early acute respiratory distress syndrome according to antiderecruitment strategy, etiological category of diffuse lung injury, and body position of the patient
7. Efficacy of lung recruiting maneuvers: It's all relative
8. Pediatric ARDS: Effect of supine-prone postural changes on oxygenation
9. Gas exchange in the ventilated patient
10. Statement of the 4th International Consensus Conference in critical care on ICU-acquired pneumonia - Chicago, Illinois, May 2002
11. Patient positioning and kinetic therapy in the critically ill patient: Pathophysiology, methods and indications
12. Optimizing intrapulmonary perfluorocarbon distribution: Fluoroscopic comparison of mode of ventilation and body position

**Records screened n=220**

**Records excluded as not relevant n= 183 (highlighted in grey)**

1. Effects of changes in trunk inclination on ventilatory efficiency in ARDS patients: quasi-experimental study.

2. Facilitated Intubation: Time to Re-examine an Old Technique With Its Associated Risks Mitigated by New Technology

3. Ventilation Distribution During Changes in Trunk Inclination in Patients With ARDS

4. The impact of semi-upright position on severity of sleep disordered breathing in patients with obstructive sleep apnea: a two-arm, prospective, randomized controlled trial

5. Curiosity, Opportunity, and Luck: Were the 1970s Different?

6. PEEP Titration Is Markedly Affected by Trunk Inclination in Mechanically Ventilated Patients with COVID-19 ARDS: A Physiologic, Cross-Over Study

7. Personalized ventilatory strategy based on lung recruitablity in COVID-19-associated acute respiratory distress syndrome: a prospective clinical study

8. Real-time effects of lateral positioning on regional ventilation and perfusion in an experimental model of acute respiratory distress syndrome.

9. Changes of blood gas analysis in moderate-to-severe acute respiratory distress syndrome peatients during long-term prone position ventilation: a retrospective cohort study

10. Pressure ulcers after prone positioning in patients undergoing extracorporeal membrane oxygenation: A cross-sectional study

11. Examination of respiratory mechanics with trunk inclination in obese and non-obese ARDS patients

12. Effects of bending and prone position ventilation on respiratory and circulatory functions in patients with ARDS

13. Robust Non-Contact Monitoring of Respiratory Rate using a Depth Camera

14. Preoperative cerebral oxygenation in high-risk noncardiac surgical patients: an observational study on postoperative mortality and complications

15. Regional ventilation in spontaneously breathing COVID-19 patients during postural maneuvers assessed by electrical impedance tomography

16. Lung ultrasound to predict gas-exchange response to prone positioning in COVID-19 patients: A prospective study in pilot and confirmation cohorts

17. Physio-Metabolic Monitoring via Breath Employing Real-Time Mass Spectrometry: Importance, Challenges, Potentials, and Pitfalls

18. Positive end-expiratory pressure and prone position alter the capacity of force generation from diaphragm in acute respiratory distress syndrome: an animal experiment

19. Paradoxical Positioning: Does "Head Up" Always Improve Mechanics and Lung Protection?

20. Effect of body position on the redistribution of regional lung aeration during invasive and non-invasive ventilation of COVID-19 patients.

21. Finite Element Modeling of Pulmonary Mechanics in Severe Acute Respiratory Distress Syndrome: Explaining the Inclination Angle?

22. In Vitro and In Vivo Feasibility Study for a Portable VV-ECMO and ECCO2R System.

23. Sequential lateral positioning as a new lung recruitment maneuver: an exploratory study in early mechanically ventilated Covid-19 ARDS patients.

24. Effects of Trunk Inclination on Respiratory Mechanics in Patients with COVID-19-associated Acute Respiratory Distress Syndrome: Let's Always Report the Angle!

25. Prolonged Continuous Monitoring of Regional Lung Function in Infants with Respiratory Failure.

26. The protective versus conventional ventilation during laparoscopic surgery trial

27. HFNC Versus Oxygen Face Mask on Postoperative Pulmonery Complications

28. Effect of sufentanil in saddle block on delivery parameters

29. Smartphone-Guided Self-prone Positioning vs Usual Care in Nonintubated Hospital Ward Patients With COVID-19: a Pragmatic Randomized Clinical Trial

30. Effect of a FLUid Bolus or a Low Dose VAsopressor Infusion on Cardiovascular Collapse Among Critically Ill Adults Undergoing Tracheal Intubation

31. Analysis of the Effects of Humidified High Flow Nasal Oxygen Therapy Combined with Noninvasive Mechanical Ventilation on Treatment OutcomeS

32. Chest wall loading in the ICU: pushes, weights, and positions

33. Capnodynamic monitoring of lung volume and blood flow in response to increased positive end-expiratory pressure in moderate to severe COVID-19 pneumonia: an observational study

34. Effects of prone and lateral positioning alternate in high-flow nasal cannula patients with severe COVID-19

35. COVID-19-Related ARDS: Key Mechanistic Features and Treatments

36. Therapeutic benefits of proning to improve pulmonary gas exchange in severe respiratory failure: focus on fundamentals of physiology

37. Prone positioning redistributes gravitational stress in the lung in normal conditions and in simulations of oedema

38. Positioning for acute respiratory distress in hospitalised infants and children

39. Match Me If You Can: The Relationship between Ventilation and Perfusion with Position Changes in Nonhomogenous Lung Injury

40. Advanced respiratory monitoring in mechanically ventilated patients with coronavirus disease 2019- associated acute respiratory distress syndrome

41. Ventilatory Management of the Patient with Severe Obesity

42. Independent lung ventilation for the management of acute allograft rejection after single-lung transplantation for end-stage emphysema

43. Prolonged non-invasive respiratory supports in a patient with COVID-19 and severe acute hypoxemic respiratory failure: A case report

44. The Effects of Rso2 and PI Monitoring Images on the Treatment of Premature Infants Based on Deep Learning

45. Combining Non-invasive Ventilation with timed position change in the Emergency Department to improve oxygenation and outcomes in patients with COVID-19: A prospective analysis from a low resource setup

46. Perioperative Pulmonary Atelectasis: Part II. Clinical Implications

47. Effect of Position Change From the Bed to a Wheelchair on the Regional Ventilation Distribution Assessed by Electrical Impedance Tomography in Patients With Respiratory Failure.

48. Diminishing Efficacy of Prone Positioning With Late Application in Evolving Lung Injury.

49. Awake Prone Positioning in COVID-19 Suspects With Hypoxemic Respiratory Failure

50. The effects of different body positions during ventilation on the cardiopulmonary function, blood gas, and inflammation indicators in severe acute respiratory distress syndrome patients (lateral position)

51. Perioperative management of emergent cesarean section in a patient with peripartum cardiomyopathy and orthopnea: a case report

52. Continuous Lower Abdominal Compression as a Therapeutic Intervention in COVID-19 ARDS

53. Platypnea–orthodeoxia syndrome associated with COVID-19 pneumonia: a case report

54. SARS-CoV-2 pneumonia succesfully treated with cpap and cycles of tripod position: a case report

55. Target and goal mismatch during mechanical ventilation in COVID-19 patients

56. Paradoxical Effect of Chest Wall Compression on Respiratory System Compliance: A Multicenter Case Series of Patients With ARDS, With Multimodal Assessment

57. A case of spontaneous rectus sheath hematoma induced by lateral semi-prone positional changes during extracorporeal membrane oxygenation

58. Effects of prone and lateral position in non-intubated patients with 2019 Novel Coronavirus (COVID-19) pneumonia

59. The Obese Patient With Acute Respiratory Failure

60. Impact of Prone Position on 12-Lead Electrocardiogram in Healthy Adults: A Comparison Study with Standard Electrocardiogram

61. How to ventilate obese patients in the ICU

62. A primer on proning in the emergency department

63. Management and experience of postural placement in postoperative mechanical ventilation of newborns. **

64. [A strange case of acute respiratory failure: the platypnea-orthodeoxia syndrome].

65. Sources of variability in expiratory flow profiles during sleep in healthy young children

66. Determinants of the esophageal-pleural pressure relationship in humans.

67. Sargramostim in patients with acute hypoxic respiratory failure due to COVID-19

68. Incline Positioning in COVID-19 Patients for Improvement in Oxygen Saturation

69. Early application of prone position for management of Covid-19 patients

70. COVID-19 associated pulmonary aspergillosis

71. Treating hypoxemic patients with SARS-COV-2 pneumonia: Back to applied physiology

72. Finding best peep: A little at a time

73. Study on the extracorporeal membrane oxygenation inter-hospital transport during coronavirus disease 2019 epidemic: Based on the transport experience of 6 cases of severe H1N1 influenza virus pneumonia on extracorporeal membrane oxygenation

74. Oxygen therapy delivery and body position effects measured with electrical impedance tomography. (spontaneous breathing)

75. The effects of forward head posture on expiratory muscle strength in chronic neck pain patients: A cross-sectional study

76. Effect of body position and inclination in supine and prone position on respiratory mechanics in acute respiratory distress syndrome.

77. Early Use of Prone Position in ECMO for Severe ARDS

78. Pharmacodynamic interaction of remifentanil and dexmedetomidine on depth of sedation and tolerance of laryngoscopy

79. Extreme obesity—particular aspect of invasive and noninvasive ventilation | Adipositas (permagna) – Besonderheiten bei der invasiven und nichtinvasiven Beatmung

80. Airway Closure during Surgical Pneumoperitoneum in Obese Patients

81. Sepsis-induced heparin resistance during extracorporeal membrane oxygenation

82. ARDS in Obese Patients: Specificities and Management

83. Building on the Shoulders of Giants: Is the use of Early Spontaneous Ventilation in the Setting of Severe Diffuse Acute Respiratory Distress Syndrome Actually Heretical?

84. Effects of High-Flow Nasal Cannula on End-Expiratory Lung Impedance in Semi-Seated Healthy Subjects.

85. The obesity supine death syndrome (OSDS).

86. Intraoperative Ventilation of Morbidly Obese Patients Guided by Transpulmonary Pressure

87. Oral levosimendan in ALS: the REFALS phase 3 study design

88. Effect of Inspiratory Muscle Training and Early Mobilization Program on Weaning of Mechanical Ventilation in Critically Ill Patients

89. CO2 Clearance During Noninvasive Ventilation (NIV)

90. Expiratory Flow Limitation During Mechanical Ventilation

91. Outcomes of extracorporeal membrane oxygenation in adult patients with hypoxemic respiratory failure refractory to mechanical ventilation

92. Electrical impedance tomography as possible guidance for individual positioning of patients with multiple lung injury (spontaneous breathing)

93. A Quasi-Experimental, Before-After Trial Examining the Impact of an Emergency Department Mechanical Ventilator Protocol on Clinical Outcomes and Lung-Protective Ventilation in Acute Respiratory Distress Syndrome

94. Lung-Protective Ventilation Initiated in the Emergency Department (LOV-ED): a Quasi-Experimental, Before-After Trial

95. The evaluation of the astral ivaps autoepap treatment algorithm

96. Extracorporeal membrane oxygenation in spina bifida and (H1N1)-induced acute respiratory distress syndrome

97. Should we use driving pressure to set tidal volume?

98. [What Should We Know about Respiratory Physiology for the Optimal Anesthesia Management?]

99. A phase IV, single-center, crossover evaluation of the efficacy of an external nasal dilator strip in children with nasal congestion.

100. Lateral positioning for critically ill adult patients.

101. Head-of-bed elevation improves end-expiratory lung volumes in mechanically ventilated subjects: a prospective observational study. Respir Care

102. The effect of sugammadex on respiratory complications and patient satisfaction following surgery

103. HFCWO on Pneumonic Respiratory Failure

104. Effect of exercise on vital capacity in different posture of young Indian subjects

105. Biotrauma and Ventilator-Induced Lung Injury: Clinical Implications

106. Mechanisms of orthopnea in stable obese subjects

107. Should we embrace the open lung approach?

108. Preoxygenation and general anesthesia: a review.

109. Short Term Physiological Effects of Nasal High Flow Oxygen on Respiratory Mechanics

110. Lung Volume Recruitment in Neuromuscular Disease: can ‘breath-stacking’ improve lung function, respiratory symptoms and quality of life for people with neuromuscular disease?

111. S2e guideline: positioning and early mobilisation in prophylaxis or therapy of pulmonary disorders: Revision 2015: S2e guideline of the German Society of Anaesthesiology and Intensive Care Medicine (DGAI) | S2e-Leitlinie

112. Advantages of the prone position for minimally invasive esophagectomy in comparison to the left decubitus position: better oxygenation after minimally invasive esophagectomy

113. A comprehensive review of prone position in ARDS

114. Awake’ extracorporeal membrane oxygenation requires adequate lower body muscle training and mobilisation as successful bridge to lung transplant

115. A comparison of supine and prone positioning on improves arterial oxygenation in premature neonates.

116. Initiation of home mechanical ventilation at home in a selectve group of patients with chronic hypercapnic respiratory failure in the Netherlands

117. 28th Annual Meeting of the European Association of Cardiothoracic Anaesthesiologists, EACTA 2013

118. Ischemic preconditioning eliminates positional changes in oxygen saturation during hypoxia

119. Should we prone cardiac surgery patients with acute respiratory distress syndrome?

120. Body position and oxygenation: An intriguing relationship

121. Sleep and breathing

122. Positioning in mechanical ventilation | Lagerungstherapie bei beatmeten Intensivpatienten

123. Ventilatory strategies and supportive care in acute respiratory distress syndrome.

124. Body position changes redistribute lung computed-tomographic density in patients with acute respiratory failure: impact and clinical fallout through the following 20 years.

125. Effect of different seated positions on lung volume and oxygenation in acute respiratory distress syndrome.

126. Effect of body position on ventilation distribution in ventilated preterm infants.

127. Effect of open and closed suction on Ventilator-associated Pneumonia and hemodynamic status

128. Is All on the Level? Hemodynamics during Supine versus Prone Ventilation

129. Effets de la ventilation en position verticale. À propos d'un patient en réanimation | Ventilation effects in vertical position. Case report on patient in intensive care unit

130. Effects of sitting position and applied positive end-expiratory pressure on respiratory mechanics of critically ill obese patients receiving mechanical ventilation

131. Erratum: Effect of different seated positions on lung volume and oxygenation in acute respiratory distress syndrome

132. Surviving sepsis campaign: International guidelines for management of severe sepsis and septic shock, 2012

133. High-frequency oscillatory ventilation

134. Acute respiratory distress syndrome

135. Platypnea-orthodeoxia syndrome related to right hemidiaphragmatic elevation and a 'stretched' patent foramen ovale.

136. Continuous lateral rotational therapy and systemic inflammatory response in posttraumatic acute lung injury: Results from a prospective randomised study

137. Perioperative management of the severely obese patient: A selective pathophysiological review

138. Relationship between regional lung compliance and ventilation homogeneity in the supine and prone position

139. Management of the critically ill obstetric patient

140. Left tilt position for easy extracorporeal membrane oxygenation cannula insertion in late pregnancy patients

141. Left tilt position for cardiopulmonary bypass in parturient patients

142. Prone positioning for patients with ARDS: Although not common in ICUs, this therapy may help improve oxygenation. Here's what you need to know

143. Positioning for acute respiratory distress in hospitalised infants and children.

144. Evident-based nursing of a ventilation patient with acute respiratory distress syndrome: Exploring a reasonable position

145. Early detection of deteriorating ventilation: Prevention is better than cure!

146. Short-term effects of combining upright and prone positions in patients with ARDS: a prospective randomized study.

147. Positioning of patients with acute respiratory distress syndrome: Combining prone and upright makes sense

148. Acute respiratory distress syndrome and acute lung injury

149. Physiology of gas exchange during anaesthesia

150. Does positive end-expiratory pressure improve CO2 exchange in controlled ventilation of acute airflow obstruction?

151. The effect of different positioning after lung recruitment with CPAP hold maneuver on oxygenation and lung mechanics in acute respiratory distress syndrome

152. Prone positioning improves survival in severe ARDS: a pathophysiologic review and individual patient meta-analysis

153. Severe hypoxemic respiratory failure: Part 2-nonventilatory strategies

154. Abdominal volume contribution to tidal volume as an early indicator of respiratory impairment in Duchenne muscular dystrophy

155. Intra-abdominal hypertension, prone ventilation, and abdominal suspension

156. Prone positioning in patients with acute respiratory distress syndrome - In reply [2]

157. Prevalence and prognosis of shunting across patent foramen ovale during acute respiratory distress syndrome

158. Pharmacotherapy for prevention and treatment of acute respiratory distress syndrome: Current and experimental approaches

159. Upright position mechanical ventilation: an alternative strategy for ALI/ARDS patients?

160. Effect of early prone postioning on mortality in patients with severe and persistent Acute Respiratory Distress Syndrome. Proseva Study. Description of the research protocol

161. Ventilating ARDS beyond PEEP and Tidal Volume

162. Effect of intrapulmonary percussive ventilation on expiratory flow limitation in chronic obstructive pulmonary disease patients

163. Functional residual capacity measurements in ventilated patients: Measures, determinants, clinical implications and perspectives

164. Extended prone position ventilation in severe acute respiratory distress syndrome: A pilot feasibility study

165. Prone positioning in hypoxemic respiratory failure: Meta-analysis of randomized controlled trials

166. Successful treatment of severe hepatopulmonary syndrome with a sequential use of TIPS placement and liver transplantation

167. Surgical techniques: Lung transplant and lung volume reduction

168. Nasal reflexes: Implications for exercise, breathing, and sex

169. Effect of recruitment and body positioning on lung volume and oxygenation in acute lung injury model

170. BTS guideline for emergency oxygen use in adult patients

171. Effects of prone position and positive end-expiratory pressure on lung perfusion and ventilation

172. Inhaled nitric oxide and prone position: how far they can improve oxygenation in pediatric patients with acute respiratory distress syndrome?

173. Prone position in early and severe acute respiratory distress syndrome: a design for a definitive randomized controlled trial

174. Association of body position with sleep architecture and respiratory disturbances in children with obstructive sleep apnea

175. Pneumomediastinum complicated by subclavian central venous catheterization in a severe thoracic trauma patient

176. Prolonged lateral steep position impairs respiratory mechanics during continuous lateral rotation therapy in respiratory failure

177. Cardiopulmonary interaction in heart failure

178. The effect of lateral position on oxygenation in ARDS patients: a pilot study

179. Effects of vertical positioning on gas exchange and lung volumes in acute respiratory distress syndrome.

180. Cardiopulmonary interaction in heart failure

181. Ventilation in the prone position in patients with acute lung injury/acute respiratory distress syndrome

182. Clinical trial design--effect of prone positioning on clinical outcomes in infants and children with acute respiratory distress syndrome

183. Mechanical ventilation in the management of acute respiratory distress syndrome

184. Acute Lung Injury and the Acute Respiratory Distress Syndrome: Challenges in Clinical Trial Design

185. Positional therapy and polytrauma: Useful or dangerous? | Lagerungstherapie beim polytrauma: Sinnvoll oder gefährlich?

186. Lateral decubitus position generates discomfort and worsens lung function in chronic heart failure.

187. [Platypnea-orthodeoxia syndrome, atrial septal aneurysm and right hemidiaphragmatic elevation with a right-to-left shunt through a patent foramen ovale].

188. Effect of body position on gas exchange in patients with idiopathic pulmonary alveolar proteinosis: no benefit of prone positioning.

189. Acute effects of upright position on gas exchange in patients with acute respiratory distress syndrome.

190. Effect of prone position on lung surfactant composition and function in multiple trauma patients with respiratory dysfunction

191. Protective lung strategies during artificial ventilation in children

192. Large venous air embolism in the sitting position despite monitoring with transoesophageal echocardiography

193. Lung recruitment during mechanical positive pressure ventilation in the PICU: What can be learned from the literature?

194. Prone positioning in children with ARDS: Positive reflections on a negative clinical trial

195. Prone positioning: Do not turn it off!

196. Positioning for acute respiratory distress in hospitalised infants and children.

197. The role of body position and gravity in the symptoms and treatment of various medical diseases.

198. The effect of various body positions on dynamic intrinsic PEEP during pressure support ventilation | Wielkość dynamicznego PEEP w czasie zmiany pozycji ciała u chorych w trakcie wentylacji wspomaganej

199. Comparison of incomplete (135°) and complete prone position (180°) in patients with acute respiratory distress syndrome. Results of a prospective, randomised trial | Vergleich von inkompletter (135°) und kompletter bauchlage (180°) beim schweren akuten lungenversagen. Ergebnisse einer prospektiven, randomisierten untersuchung

200. Acute respiratory distress syndrome

201. Comparison of positive end-expiratory pressure with reverse Trendelenburg position in morbidly obese patients undergoing bariatric surgery: effects on hemodynamics and pulmonary gas exchange.

202. Effect of alveolar recruitment maneuver in early acute respiratory distress syndrome according to antiderecruitment strategy, etiological category of diffuse lung injury, and body position of the patient.

203. The upper airway in pregnancy and pre-eclampsia.

204. ARDS management: high-frequency oscillatory ventilation as a new option

205. Evidence-based medicine in ARDS: what is really proven?

206. Decrease in Paco2 with prone position is predictive of improved outcome in acute respiratory distress syndrome

207. Use of orthostasis during artificial pulmonary ventilation in patients with acute parenchymal pulmonary lesion

208. Prone positioning of pediatric patients with ARDS results in improvement in oxygenation if maintained > 12 h daily

209. Combined effects of prone positioning and airway pressure release ventilation on gas exchange in patients with acute lung injury

210. Acute respiratory distress syndrome: Lessons from computed tomography of the whole lung

211. Efficacy of lung recruiting maneuvers: It's all relative

212. Effect of alveolar recruitment maneuver in early acute respiratory distress syndrome according to antiderecruitment strategy, etiological category of diffuse lung injury, and body position of the patient

213. Efficacy of lung recruiting maneuvers: It's all relative

214. Pediatric ARDS: Effect of supine-prone postural changes on oxygenation

215. Gas exchange in the ventilated patient

216. A randomized trial on the effects of body positions on lung function with acute respiratory failure patients

217. Statement of the 4th International Consensus Conference in critical care on ICU-acquired pneumonia - Chicago, Illinois, May 2002

218. Patient positioning and kinetic therapy in the critically ill patient: Pathophysiology, methods and indications

219. Optimizing intrapulmonary perfluorocarbon distribution: Fluoroscopic comparison of mode of ventilation and body position.

220. Impact of Supine Versus Semirecumbent Body Posture on the Distribution of Ventilation in Acute Respiratory Distress Syndrome. Crit Care Explor. 2023 Dec 1;5(12):e1014.

**Studies assessed for eligibility n = 37**

**Studies were excluded from the search strategy for not meeting the inclusion criteria or presenting exclusion criteria. N = 24 (highlighted in grey)**

1. Effects of changes in trunk inclination on ventilatory efficiency in ARDS patients: quasi-experimental study.
2. Ventilation Distribution During Changes in Trunk Inclination in Patients With ARDS
3. PEEP Titration Is Markedly Affected by Trunk Inclination in Mechanically Ventilated Patients with COVID-19 ARDS: A Physiologic, Cross-Over Study
4. The impact of semi-upright position on severity of sleep disordered breathing in patients with obstructive sleep apnea: a two-arm, prospective, randomized controlled trial
5. Examination of respiratory mechanics with trunk inclination in obese and non-obese ARDS patients
6. Paradoxical Positioning: Does "Head Up" Always Improve Mechanics and Lung Protection?
7. Effect of body position on the redistribution of regional lung aeration during invasive and non-invasive ventilation of COVID-19 patients.
8. Finite Element Modeling of Pulmonary Mechanics in Severe Acute Respiratory Distress Syndrome: Explaining the Inclination Angle?
9. Effects of Trunk Inclination on Respiratory Mechanics in Patients with COVID-19-associated Acute Respiratory Distress Syndrome: Let's Always Report the Angle!
10. Effect of Position Change From the Bed to a Wheelchair on the Regional Ventilation Distribution Assessed by Electrical Impedance Tomography in Patients With Respiratory Failure
11. The effects of different body positions during ventilation on the cardiopulmonary function, blood gas, and inflammation indicators in severe acute respiratory distress syndrome patients
12. Incline Positioning in COVID-19 Patients for Improvement in Oxygen Saturation
13. The effects of forward head posture on expiratory muscle strength in chronic neck pain patients: A cross-sectional study
14. Effect of body position and inclination in supine and prone position on respiratory mechanics in acute respiratory distress syndrome.
15. Electrical impedance tomography as possible guidance for individual positioning of patients with multiple lung injury (spontaneous breathing)
16. Body position and oxygenation: An intriguing relationship
17. Positioning in mechanical ventilation | Lagerungstherapie bei beatmeten Intensivpatienten
18. Body position changes redistribute lung computed-tomographic density in patients with acute respiratory failure: impact and clinical fallout through the following 20 years.
19. Effect of different seated positions on lung volume and oxygenation in acute respiratory distress syndrome.
20. Effect of body position on ventilation distribution in ventilated preterm infants.
21. Effets de la ventilation en position verticale. À propos d'un patient en réanimation | Ventilation effects in vertical position. Case report on patient in intensive care unit
22. Effects of sitting position and applied positive end-expiratory pressure on respiratory mechanics of critically ill obese patients receiving mechanical ventilation
23. Erratum: Effect of different seated positions on lung volume and oxygenation in acute respiratory distress syndrome
24. Short-term effects of combining upright and prone positions in patients with ARDS: a prospective randomized study.
25. Head-of-bed elevation improves end-expiratory lung volumes in mechanically ventilated subjects: a prospective observational study. Respir Care
26. Positioning of patients with acute respiratory distress syndrome: Combining prone and upright makes sense
27. Upright position mechanical ventilation: an alternative strategy for ALI/ARDS patients?
28. Effect of recruitment and body positioning on lung volume and oxygenation in acute lung injury model
29. Effects of vertical positioning on gas exchange and lung volumes in acute respiratory distress syndrome
30. Effect of body position on gas exchange in patients with idiopathic pulmonary alveolar proteinosis: no benefit of prone positioning.
31. Acute effects of upright position on gas exchange in patients with acute respiratory distress syndrome.
32. The role of body position and gravity in the symptoms and treatment of various medical diseases.
33. The effect of various body positions on dynamic intrinsic PEEP during pressure support ventilation | Wielkość dynamicznego PEEP w czasie zmiany pozycji ciała u chorych w trakcie wentylacji wspomaganej
34. Comparison of positive end-expiratory pressure with reverse Trendelenburg position in morbidly obese patients undergoing bariatric surgery: effects on hemodynamics and pulmonary gas exchange.
35. Effect of alveolar recruitment maneuver in early acute respiratory distress syndrome according to antiderecruitment strategy, etiological category of diffuse lung injury, and body position of the patient.
36. A randomized trial on the effects of body positions on lung function with acute respiratory failure patients
37. Impact of Supine Versus Semirecumbent Body Posture on the Distribution of Ventilation in Acute Respiratory Distress Syndrome. Crit Care Explor. 2023 Dec 1;5(12):e1014.

Studies excluded:

1. Patients on spontaneous ventilation (N=5)

- The impact of semi-upright position on severity of sleep disordered breathing in patients with obstructive sleep apnea: a two-arm, prospective, randomized controlled trial
- Effect of Position Change From the Bed to a Wheelchair on the Regional Ventilation Distribution Assessed by Electrical Impedance Tomography in Patients With Respiratory Failure
- Incline Positioning in COVID-19 Patients for Improvement in Oxygen Saturation
- The effects of forward head posture on expiratory muscle strength in chronic neck pain patients: A cross-sectional study
- Electrical impedance tomography as possible guidance for individual positioning of patients with multiple lung injury)

1. Patients in lateral or prone positioning (N=8)

- Effect of body position on the redistribution of regional lung aeration during invasive and non-invasive ventilation of COVID-19 patients
- The effects of different body positions during ventilation on the cardiopulmonary function, blood gas, and inflammation indicators in severe acute respiratory distress syndrome patients
- Effect of body position on ventilation distribution in ventilated preterm infants.
- Short-term effects of combining upright and prone positions in patients with ARDS: a prospective randomized study.
- Effect of recruitment and body positioning on lung volume and oxygenation in acute lung injury model
- Effect of body position on gas exchange in patients with idiopathic pulmonary alveolar proteinosis: no benefit of prone positioning.
- Effect of alveolar recruitment maneuver in early acute respiratory distress syndrome according to antiderecruitment strategy, etiological category of diffuse lung injury, and body position of the patient.
- A randomized trial on the effects of body positions on lung function with acute respiratory failure patients

1. Editorial (N= 3)

- Finite Element Modeling of Pulmonary Mechanics in Severe Acute Respiratory Distress Syndrome: Explaining the Inclination Angle?
- Positioning of patients with acute respiratory distress syndrome: combining prone and upright makes sense
- Upright position mechanical ventilation: an alternative strategy for ALI/ARDS patients?

1. Case Report (N=2)

- Body position and oxygenation: An intriguing relationship
- Effets de la ventilation en position verticale. À propos d'un patient en réanimation | Ventilation effects in vertical position. Case report on patient in intensive care unit

1. Review (N=2)

- Body position changes redistribute lung computed-tomographic density in patients with acute respiratory failure: impact and clinical fallout through the following 20 years.
- The role of body position and gravity in the symptoms and treatment of various medical diseases.

1. Error in the editing of the study. (N=1)

- Erratum: Effect of different seated positions on lung volume and oxygenation in acute respiratory distress syndrome

1. Not written in the English language (N=2)

- The effect of various body positions on dynamic intrinsic PEEP during pressure support ventilation | Wielkość dynamicznego PEEP w czasie zmiany pozycji ciała u chorych w trakcie wentylacji wspomaganej
- Positioning in mechanical ventilation | Lagerungstherapie bei beatmeten Intensivpatienten

1. Surgical patients without acute respiratory failure (N=1)

- Comparison of positive end-expiratory pressure with reverse Trendelenburg position in morbidly obese patients undergoing bariatric surgery: effects on hemodynamics and pulmonary gas exchange.

***Studies included in scoping review (n=13)***

1. Effects of changes in trunk inclination on ventilatory efficiency in ARDS patients: quasi-experimental study. (Benites et al.)
2. Ventilation Distribution During Changes in Trunk Inclination in Patients With ARDS (Marrazzo et al.)
3. PEEP Titration Is Markedly Affected by Trunk Inclination in Mechanically Ventilated Patients with COVID-19 ARDS: A Physiologic, Cross-Over Study (Marrazzo et al.)
4. Examination of respiratory mechanics with trunk inclination in obese and non-obese ARDS patients (Shailesh Bihari et al.)
5. Paradoxical Positioning: Does "Head Up" Always Improve Mechanics and Lung Protection? (Selickman et al.)
6. Effects of Trunk Inclination on Respiratory Mechanics in Patients with COVID-19-associated Acute Respiratory Distress Syndrome: Let's Always Report the Angle! (Marrazzo et al.)
7. Effect of different seated positions on lung volume and oxygenation in acute respiratory distress syndrome. (Dellamonica et al.)
8. Effect of body position and inclination in supine and prone position on respiratory mechanics in acute respiratory distress syndrome. (Mezidi et al.)
9. Effects of sitting position and applied positive end-expiratory pressure on respiratory mechanics of critically ill obese patients receiving mechanical ventilation (Lemyze et al.)
10. Head-of-bed elevation improves end-expiratory lung volumes in mechanically ventilated subjects: a prospective observational study. (Spooner et al.)
11. Effects of vertical positioning on gas exchange and lung volumes in acute respiratory distress syndrome (Richard et al.)
12. Acute effects of upright position on gas exchange in patients with acute respiratory distress syndrome ( Hoste et al.)
13. Impact of Supine Versus Semirecumbent Body Posture on the Distribution of Ventilation in Acute Respiratory Distress Syndrome. Crit Care Explor. 2023 Dec 1;5(12):e1014.
