# Supplementary material for: Physiological and clinical effects of trunk inclination adjustment in patients with respiratory failure: a scoping review and narrative synthesis
Source: Crit Care. 2024 Jul 9;28:228. doi: 10.1186/s13054-024-05010-1 (PMC11232125; doi:10.1186/s13054-024-05010-1)
Supplement: Supplementary file 2 — Additional file 2. [file 13054_2024_5010_MOESM2_ESM.docx]

**Physiological and clinical effects of trunk inclination adjustment in patients with respiratory failure. A Scoping review and narrative synthesis**

**Additional file 2**

Table: The risk of bias was assessed using the Quality Assessment Tool for Before-After (Pre-Post) Studies with No Control Group developed by NHLBI of the US NIH.

|  | Hoste et al. J Intensive Care Med 2005 | Richard et al. Intensive Care Med 2006 | Delllamonica et al. Intensive Care Med 2013 | Lemyze et al. Crit Care Med. 2013 | Spooner at al. Respir Care 2014 | Midizzi et al. Intensive Care Med 2019 | Marrazzo et al. Am J Respir Crit Care Med 2022 | Selickman et al. Crit Care Med 2022 | Marrazzo et al. Journal of clinical medicine 2023 | Marrazzo et al. Respir Care. 2023 | Benites et al. Intensive Care Med Experimental 2023 | Bihari et al. Chest 2023 | Pearce et al.  Crit Care Explor 2023 |
| --- | --- | --- | --- | --- | --- | --- | --- | --- | --- | --- | --- | --- | --- |
| 1. Was the study question or objective clearly stated? | yes | yes | yes | yes | yes | yes | yes | yes | yes | yes | yes | yes | yes |
| 2. Were eligibility/selection criteria for the study population prespecified and clearly described? | yes | no | yes | yes | yes | no | yes | yes | yes | yes | yes | yes | yes |
| 3. Were the participants in the study representative of those who would be eligible for the test/service/intervention in the general or clinical population of interest? | yes | yes | yes | yes | yes | yes | yes | yes | yes | yes | yes | yes | yes |
| 4. Were all eligible participants that met the prespecified entry criteria enrolled? | not reported | not reported | not reported | not reported | not reported | not reported | not reported | not reported | not reported | not reported | not reported | not reported | not reported |
| 5. Was the sample size sufficiently large to provide confidence in the findings? | not reported | not reported | not reported | not reported | yes | not reported | not reported | not reported | not reported | yes | yes | not reported | not reported |
| 6. Was the test/service/intervention clearly described and delivered consistently across the study population? | yes | yes | yes | yes | yes | yes | yes | yes | yes | yes | yes | yes | yes |
| 7. Were the outcome measures prespecified, clearly defined, valid, reliable, and assessed consistently across all study participants? | not reported | not reported | not reported | not reported | not reported | not reported | not reported | not reported | not reported | not reported | yes | not reported | not reported |
| 8. Were the people assessing the outcomes blinded to the participants' exposures/interventions? | not applicable | not applicable | not applicable | not applicable | not applicable | not applicable | not applicable | not applicable | not applicable | not applicable | not  applicable | not applicable | not applicable |
| 9. Was the loss to follow-up after baseline 20% or less? Were those lost to follow-up accounted for in the analysis? | not reported | not reported | not reported | not reported | not reported | not reported | not reported | not reported | not reported | not reported | not  reported | not reported | not reported |
| 10. Did the statistical methods examine changes in outcome measures from before to after the intervention? Were statistical tests done that provided p values for the pre-to-post changes? | yes | yes | yes | yes | yes | yes | yes | yes | yes | yes | yes | yes | yes |
| 11. Were outcome measures of interest taken multiple times before the intervention and multiple times after the intervention (i.e., did they use an interrupted time-series design)? | yes | yes | yes | yes | yes | yes | yes | yes | yes | yes | yes | yes | yes |
| 12. If the intervention was conducted at a group level (e.g., a whole hospital, a community, etc.) did the statistical analysis take into account the use of individual-level data to determine effects at the group level? | not applicable | not applicable | not applicable | not applicable | not applicable | not applicable | not applicable | not applicable | not applicable | not applicable | not  applicable | not applicable | not applicable |
| Risk of bias | moderate | moderate | moderate | moderate | low | moderate | moderate | moderate | moderate | low | low | moderate | moderate |

None of the studies explicitly mentioned conducting a patient screening log process, nor did they confirm that all eligible subjects were enrolled in the study. Given that the intervention in all cases involved changing the bed inclination angle for mechanically ventilated patients, none of the studies could perform a blinded assessment of outcomes concerning participant exposure or intervention.
